# A Review of Sensory Feedback in Upper-Limb Prostheses From the Perspective of Human Motor Control

**DOI:** 10.3389/fnins.2020.00345

**Published:** 2020-06-23

**Authors:** Jonathon W. Sensinger, Strahinja Dosen

**Affiliations:** ^1^Institute of Biomedical Engineering, University of New Brunswick, Fredericton, NB, Canada; ^2^Department of Health Science and Technology, The Faculty of Medicine, Integrative Neuroscience, Aalborg University, Aalborg, Denmark

**Keywords:** prostheses, sensory feedback, computational motor control, sensory integration, human–machine interfaces

## Abstract

This manuscript reviews historical and recent studies that focus on supplementary sensory feedback for use in upper limb prostheses. It shows that the inability of many studies to speak to the issue of meaningful performance improvements in real-life scenarios is caused by the complexity of the interactions of supplementary sensory feedback with other types of feedback along with other portions of the motor control process. To do this, the present manuscript frames the question of supplementary feedback from the perspective of computational motor control, providing a brief review of the main advances in that field over the last 20 years. It then separates the studies on the closed-loop prosthesis control into distinct categories, which are defined by relating the impact of feedback to the relevant components of the motor control framework, and reviews the work that has been done over the last 50+ years in each of those categories. It ends with a discussion of the studies, along with suggestions for experimental construction and connections with other areas of research, such as machine learning.

## Introduction

Anyone who has tried to light a match with cold, numb fingers can appreciate the role that somatosensory feedback plays in accomplishing tasks. And yet although sensory feedback is important, it is only one piece of a complicated story. Cold numb fingers impact both the sensations and the control of finger movements. Small delicate tasks may be influenced by sensory deficits in ways that larger, gross motions would not. And it is possible that one would learn to compensate for numb fingers over time (say after a surgically induced numbing) such that it was only a minor inconvenience, relying on training, experience, and alternative sensory cues (e.g., visual observation). A particularly illustrative example is a well-known deafferented patient Ian Waterman, who was able, after extensive and tedious training, to grasp and manipulate objects despite having completely lost the sense of touch and proprioception (BBC, 1998; Hermsdörfer et al., 2008). Sensory feedback is indeed important, but it is part of a complicated, multifaceted system that makes it difficult to assess the true value and limitations of individual sensory percepts when used to supplement systems with sensory deficits such as prostheses.

Sensory feedback in prostheses is presently a hot topic in research, with the number of studies increasing dramatically over the past few years presenting invasive (Pasluosta et al., 2018) as well as non-invasive solutions (Svensson et al., 2017) (see Appendix for chronological list). In addition, prosthesis companies show an increasing interest in the topic (e.g., https://vincentsystems.de/en/prosthetics/vincent-evolution-2/, http://www.psyonic.co/abilityhand). However, this “boom” is not in any way unique. Something similar happened decades ago, in the 1970’s and 1980’s. In fact, in 1980 D. Childress wrote a review on sensory feedback in prosthetics from a “historical perspective” (Childress, 1980). The literature from that period is rich, and the manuscripts present methods and prototypes that are in many cases analogous to those that are being developed today. For example, an interested reader can find solutions based on electro (Shannon, 1979; Scott et al., 1980) and vibrotactile stimulation (Shannon, 1976), force applicators (Meek et al., 1989) as well as pressure cuffs (Patterson and Katz, 1992). Yet none of these solutions has been translated into clinical use.

A plausible explanation for this failure to clinically endure could be that the technology of that time was simply not mature enough to be suitable for clinical applications. Since the technology developed immensely in the meantime, one can be far more optimistic that the recent research efforts will indeed lead to a solution that will be accepted and used outside of research labs. However, once the recent literature is carefully examined, the optimism can be tainted by a doubt; the reports in the literature on the benefits of feedback are contradictory. Some studies report that the feedback significantly improves prosthesis performance (Clemente et al., 2016, 2019), whereas the others find no difference in prosthesis performance with and without feedback (Cipriani et al., 2008; Saunders and Vijayakumar, 2011), or report that the feedback is useful in only some subjects and conditions (Chatterjee et al., 2008; Markovic et al., 2018a). And indeed, both authors of the present manuscript experienced the elusiveness of prosthesis feedback when they started working on the topic several years ago. At that time, they designed their first feedback systems (independently from each other) and enthusiastically tested them in amputees, successfully demonstrating that the subjects could accomplish delicate tasks using a sensate prosthesis. However, the excitement was soon replaced by surprise, when the subjects performed the very same tasks equally well without the supplementary feedback.

The thesis of this manuscript is accordingly that the lack of feedback in commercial prostheses is not only due to deficient technology, but also at least in part due to insufficient knowledge and understanding about the fundamental role of feedback in prosthesis control. Our aim here is to shed light on some of these aspects by placing the feedback within the broader framework of human motor control.

This paper attempts to tackle the aspect of sensory feedback in prostheses, which is an integral part of a larger system of prosthetic control. For a holistic overview of prosthesis control in the broader domain, see Sensinger et al. (2019). For an overview focusing explicitly on control and feedback, see Micera et al. (2010).

Several reviews have been published recently on the topic of sensory feedback in prosthetics (Antfolk et al., 2013c; Schofield et al., 2014; Svensson et al., 2017); while they thoroughly describe the technology and methods to elicit tactile sensations, the present manuscript has a different focus. The primary purpose of this paper is to supply a lexicon – and through it a paradigm shift – in how we view the complex phenomenon of closed-loop control of myoelectric prostheses. Our lexicon and paradigm are founded in the language of computational motor control – a field that has proved influential in the broader motor control community to make sense of the way humans move. Therefore, we begin by providing an overview of the main concepts in computational motor control and relate those concepts to the realm of closed-loop prosthesis control. The secondary purpose of this paper is to supply a roadmap that explains how the various aspects work together, and how the literature has landed on the map. To this aim, we provide a comprehensive review of the state-of-the-art and organize the studies using an original categorization that reflects the computational motor control perspective. We then suggest how this roadmap may be used to remember the factors that are important to consider/control/report when experimentally assessing the effectiveness of feedback. Finally, we conclude the paper by discussing psychological factors, emerging and future work as well as connections with other research areas.

## Motor Control

### Motivation

Human movement is coordinated and consistent even within its diversity. These properties have been well known for many years, and are well posed in the pioneering work of Bernstein (Bernstein, 1967). Over the last 70 years scientists and engineers have sought to construct normative laws that describe the “what,” “how,” and “why” of human movement. These three concepts are formalized in Marr’s terminology (Marr, 1982), which divides the three questions into physical, algorithmic, and computational levels. Table 1 depicts the application of Marr’s terminology to the field of closed-loop prosthesis control. Physical and algorithmic levels are dependent on the specific properties of the system – such as the type of prosthetic control, or the fidelity and type of feedback available – whereas the computational level seeks to explain the driving purpose and logic of actions, and thus transcends specific devices. It is accordingly useful to have a clear computational framework when discussing recent advances in specific physical and algorithmic prosthetic solutions, as the computational language can transcend individual technologies. It is the aim of the present manuscript to introduce such a computational framework in the context of closed-loop prosthesis control.

**TABLE 1:** Levels of modeling classification, using levels of Marr, applied to the context of supplementary feedback in upper-limb prostheses.

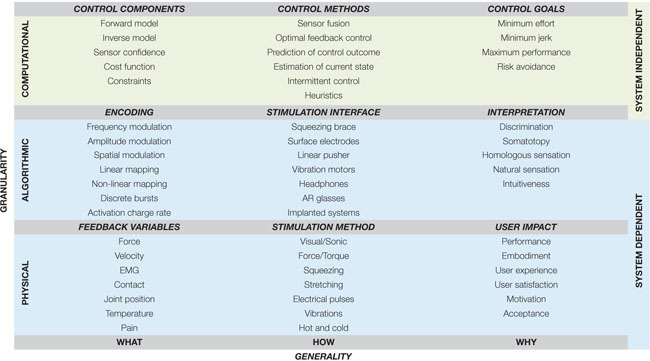

*Note that the computational layer, which is the focus of the present manuscript, is not directly dependent on the specifics of a concrete system (layers 1 and 2). Visualization adapted from Schrater et al. (2019).*

### Overview of Computational Motor Control

Human movement is regular – particularly when viewed from an appropriate framework. Through history, paradigm shifts in how we understand human movement have progressed to better explain diverse motor control, while favoring simple, elegant frameworks (Fitts, 1954; Flash and Hogan, 1985; Uno et al., 1989; Harris and Wolpert, 1998; Guiard and Beaudouin-Lafon, 2004; Soukoreff and MacKenzie, 2004). Variability is an inherent aspect of human movement that impacts the types of stereotypical movements humans make. There is substantial stochastic noise in human movements, as anyone who has tried to learn to throw a ball accurately at a target can appreciate. From the control point of view, the human nervous system is an impressive controller that can cope with the noise and adapt movements in real-time, as well as across trials to achieve the desired goal. Although feedback has long been included as a mechanism within human movement paradigms, it is only within the last 20 years that it has become an intrinsic component in the motor control policy (Todorov and Jordan, 2002), and doing so has yielded substantial insight and generalizability.

A major breakthrough in the field of computational motor control came with the work of Todorov (Todorov and Jordan, 2002; Todorov, 2005) who used the mathematical language of optimal feedback control. Human motor control and sensory feedback both have multiplicative noise, meaning that the variability of the control signal increases relative to the amplitude of the signal (De Luca, 1979; Clancy et al., 2001, 2002; Jones et al., 2002). The nature of variability in control signals affects user behavior (Chhabra and Jacobs, 2006), and is accordingly important to incorporate in any computational model that seeks to explain human behaviors, including those that are relevant for prosthesis use (as explained in later sections). Todorov was able to develop an efficient approach that captured the implications of these noise sources on many types of human behavior in an optimal control context.

Excellent overviews of the approach are provided by Scott (2004); Todorov (2004), Kording (2007), and Shadmehr and Krakauer (2008). In summary, the theory of optimal feedback control states that humans rely on the following components when controlling movements (Todorov, 2004; Shadmehr and Krakauer, 2008):

(1) Costs and rewards: at any time within a motion, there are multiple potential actions. To consider which action to take, we need to know the costs associated with each action, along with the rewarding nature of the sensory states that it may achieve. Given the stochastic nature of control, the costs and rewards are formulated as expectations, rather than as deterministic facts.
(2) Internal models: to map potential actions to the expected states they will produce (and thus the expected rewards they will incur), we need to have learned a mapping between causes (actions) and effects (anticipated state). This mapping is termed an internal model (Kawato, 1999; Cisek, 2009).
(3) Optimal feedback-driven policy: given known costs and known internal models, we need to find the optimal policy that will maximize our reward (or minimize our cost, depending on how the problem is phrased).
(4) State estimation: at every moment in time, we must estimate our state, since combining the estimate of the state with our optimal control policy will yield the control action we should take. Our estimate of state will be informed by fusing all sensory information that we have, but unfortunately, this sensory information itself has variability, and perhaps more importantly, it also has substantial delays (limiting the gains we could employ in a closed-loop framework). To compensate for this, optimal estimation blends in estimates of our effects, given knowledge of our actions and our internal model.

It seems likely that the same components govern the motor control loop of an upper limb amputee using his/her bionic limb (Johnson et al., 2017a). Therefore, to design and implement an effective closed-loop interface, it is imperative to understand how each of these elements work in an amputee equipped with a prosthesis. However, although these components have been extensively investigated in able-bodied subjects, the literature on how they work in an amputee is nascent. The emerging literature seems to generally confirm what the framework proposes – namely that the computational methods are similar between able-bodied subjects and prosthesis users [e.g., both seem to use Bayesian integration (Risso et al., 2019), internal models (Lum et al., 2014)], but that the parameters (such as control and sensory noise) are different, leading to different internal model uncertainty and ultimately different behaviors and strategies (Johnson et al., 2017a).

### Cost Functions

Cost functions define what we care about; the relationship between the quantity of that element and how much we care; and how we prioritize or weight the various things that are important to us. Humans typically care about things such as being accurate or minimizing effort (Todorov and Jordan, 2002; O’Sullivan et al., 2009). Recent work has also suggested that we aim to minimize variability and/or the amount of time a movement takes (Haith et al., 2012). Other studies have proposed we may care about making conservative movements (Nagengast et al., 2010), along with a variety of other costs, but for many upper-limb motions, considering a subset of accuracy, variability, effort, and time describes well human behavior (Shadmehr and Mussa-Ivaldi, 2012). Different relationships (e.g., linear, quadratic, exponential, and hyperbolic) have been used to model how humans penalize costs of increasing magnitudes. Mathematically, cost functions can be formulated as expressions that include these quantities and associated weights, which define the relative importance of those quantities to the human subject. The way that cost functions mathematically describe both the relative importance of small vs. large magnitudes and the relative importance of competing costs enables computational motor control models to evaluate and describe the rationale behind the choices people make when performing a movement.

These choices likely depend on the type of movement being made, as well as the unique preferences of the individual making the movement. Many studies assume quadratic cost functions because they are mathematically tractable and generally describe observed behaviors (Todorov and Jordan, 2002). A few studies have inductively assessed the actual cost functions of humans, and these studies typically find near-quadratic cost functions (Körding and Wolpert, 2004b; Sensinger et al., 2015), with the exception of time, where a hyperbolic cost function seems more representative (Shadmehr and Mussa-Ivaldi, 2012). However, these studies have only been done on a limited number of movements, and none of them have been performed on amputees (although one performed using myoelectric control found similar results, Sensinger et al., 2015). Based on the biological underpinnings of these cost functions (Haith et al., 2012; Shadmehr and Mussa-Ivaldi, 2012), it seems reasonable to assume that the cost functions are similar between able-bodied persons and those using a prosthesis. The weights between competing cost functions, however, are likely to be different across tasks, and may be different between able-bodied persons and those using a prosthesis. No work has yet explored these potential differences, although recent work has suggested flexible control solutions that adjust depending on a particular users preferences (Arunachalam et al., 2019).

Looking specifically at the contribution of supplemental feedback to improve costs, it is clear that the benefits of supplemental feedback depend on the nature and complexity of the task (Markovic et al., 2018a). There are a number of tasks in daily life, many of them included in clinical tests for prosthesis control, that can be accomplished without regulating the grip strength (Schiefer et al., 2016) (e.g., the prosthesis can be closed maximally to grasp a non-breakable object). Obviously, supplying feedback on the grasping force in such tasks is not going to contribute to the performance. Feedback is more likely useful in challenging tasks that require controlled changes of the prosthesis state (Tyler, 2016). It is accordingly useful when considering the role of supplementary feedback to explicitly identify the cost functions relevant for a given task.

### Internal Models

Internal models map the relationship between causes and effects, and they may work forward (cause to effect) or inverse (effect to cause) (Kawato, 1999; Cisek, 2009). To determine which action would produce the desired effect, humans use an inverse model. Inverse models are therefore an essential part in feedforward control, which is characteristic of learned (automatic), fast and ballistic movements. Such movements are executed by “releasing” predefined sequences of motor commands (motor programs) that were developed through experience and repeated practice. In contrast, if the aim is to predict the sensory consequence of an action before receiving the delayed sensory reading, you would use a forward internal model – also called an efference copy (Cisek, 2009). Internal models are learned from acquired feedback, but in real-time execution, they do not need feedback and indeed can even be used in place of feedback.

Internal models are important because sensory feedback is delayed. Most sensory feedback work within the realm of prostheses has assumed that supplementary feedback is useful for real-time regulation, but in reality, all sensory feedback – both intrinsic and supplementary, takes time to reach the central nervous system and be processed. This delay is on the order of 50–300 ms, and substantially limits the ability of the central nervous system to respond strongly without losing stability (Whitney, 1977). Studies have shown that for a variety of tasks, humans are able to regulate their motions and forces without any delay (Flanagan and Wing, 1997). A strong plausible explanation for this result is that they use inverse models to generate motor commands directly from the desired goal (feedforward control) and/or forward internal models to predict the effects of their actions, and then act appropriately (Kawato, 1999; Cisek, 2009). Thus, many attributes that we may assume are provided by feedback are actually subconsciously provided by our internal models.

When the predictions of our internal models are inaccurate, we update them, and there is a vast literature in this area (Shadmehr and Mussa-Ivaldi, 1994; Osu et al., 2003). It is likely that we only update them when we become confident their predictions of our state estimates are wrong (Fishbach et al., 2007). Humans can more quickly adapt their internal models when only parameters must be tuned (e.g., having mastered a badminton racquet, learning to use a tennis racquet) than when the task has different dynamics (e.g., a racquet without a handle) (Braun et al., 2009, 2010). Interestingly, human subjects are capable of updating the internal models of object dynamics after only a few grasping trials (sometimes even one) (Flanagan et al., 2001). When asked to grasp an object of unexpected weight, the subjects produce feedback corrections in load and grasp forces in the very first trial. However, the corrections fade out in later trials with the same object, indicating that the subject recalibrated the anticipatory control. Humans can accordingly update their internal models to improve future control of their motions.

A variety of studies have demonstrated the usefulness of internal models in controlling prostheses. It was shown in Lum et al. (2014) that the subjects properly scaled the grasping forces depending on the object fragility and that this scaling was refined over successive trials (inverse model adaptation). Similarly, as reported in Weeks et al. (2000), the subjects anticipatory increased the force when the weight of the object held by the prosthesis was predictably increased. However, in general, the accuracy of such internal models is poor, and the performance is variable across subjects. This is at least partially related to the uncertainty that characterizes the generation of myoelectric signals, which are imbued with multiplicative signal-dependent noise. For example, when amputees were asked to produce repeatedly the same level of grasping force, they could do that rather consistently if the target level was low but the performance decreased substantially for the high target (Ninu et al., 2014). Strengthening internal models is accordingly a clear way to improve output performance.

The usefulness of internal models vs. feedback depends on the quality and availability of inverse models. If the task is simple and control reliable, supplementary feedback might not be useful since the subjects can control their prosthesis in a purely feedforward fashion. This is nicely demonstrated in Saunders and Vijayakumar (2011), where vibrotactile force feedback did not improve grasping performance with respect to no feedback, even in a condition of full sensory deprivation. Conversely, recent research has used feedback that was specifically designed to exploit and supplement the use of internal models in amputees [see sections “Biofeedback to facilitate forward models (efference copy)” and “Delivering feedback to improve feedforward control (inverse model)”]. There is accordingly a complicated but tractable relationship between feedback and internal models as they affect each other and output performance.

The contribution of feedback is also linked to how much training participants have received. When subjects have not yet received extensive training, feedback is useful – both to develop internal models as well as to execute real-time corrections. Over time, however, as participants develop better internal models, the usefulness of feedback for real-time corrections may fade (Strbac et al., 2017; Markovic et al., 2018a). This is likely because the subjects acquire inverse models and/or learn to perceive and interpret the incidental sources of information. Feedback is not necessarily most beneficial before subjects have received any training, however, as efference copies also enhance the impact of feedback (Cuberovic et al., 2019). Prior to development of these decoding internal models, feedback has been found to be less useful; for example, as demonstrated in Markovic et al. (2018a), the subjects needed some time to learn to control a prosthesis in a delicate task before they were able to exploit the feedback successfully. In summary, the impact of feedback highly depends on how much training a participant has received to develop their inverse and forward internal models.

### Optimal Feedback Control Policies

For a given set of costs and a given set of properties, including internal models of system dynamics and estimations of sensory feedback and control stochastic noise sources, an optimal feedback control policy decides on the best course of action for a given state. In contrast to plans that assume a specific sequence of states (fixed trajectory), the policies are general rules that define optimal transitions toward the goal from any state. For example, directions to a destination is an example of a trajectory, whereas traversing the shortest distance using a map is an example of a policy.

Humans use optimal or near-optimal policies across a variety of tasks (Kording, 2007; Liu and Todorov, 2007; Shadmehr and Mussa-Ivaldi, 2012; Acerbi et al., 2014), although it is important to note that for some tasks, their decisions do not seem optimal (Shadmehr and Mussa-Ivaldi, 2012). The challenge of optimal feedback control theories is to explain human abilities to generate these optimal policies in the simplest, most efficient way possible. In a general case, the optimal policies can be derived by applying the framework of optimal control (Todorov, 2006) and dynamic programming (Bertsekas, 2014). For systems with linear dynamics and quadratic costs, this problem substantially reduces to a linear quadratic regulator, and near-optimal solutions can be found using iterative linear quadratic regulators (Li and Todorov, 2007), reinforcement learning (Kositsky and Barto, 2001; Reinkensmeyer et al., 2012), or other strategies (Todorov, 2009). Many of these approaches provide relatively simple explanations, with explanatory value such as being able to describe human movement behavior, uncontrolled manifolds and synergies, or the asymmetrical velocity profiles found in many human movements (Todorov, 2009; Mitrovic et al., 2010; Rigoux and Guigon, 2012; Shadmehr and Mussa-Ivaldi, 2012). Perhaps most importantly for the context of this paper, they provide insight into the contribution of feedback throughout the process. Similar models have only recently been applied to explain the behavior of a prosthesis user (Johnson et al., 2014, 2017a). However, this research is still in its initial phase and we have yet to develop models that can comprehensively describe the use of a prosthesis in clinically relevant situations.

Optimal feedback control policies help to explain the contribution of supplemental feedback relative to the other properties of the system. The optimal policy of a human controller takes into account the uncertainty of feedforward and feedback pathways. Therefore, the effectiveness of supplementary feedback is also affected by the quality of control. Control quality depends on both the command interface, which determines precision and accuracy in generating command signals, and on the characteristics of a controlled system, which defines the consistency of the system response to those commands. These aspects were investigated in Dosen et al. (2015b) where the subjects used less and more reliable interface (myoelectric versus joystick) to control a system with less and more consistent response (real versus simulated prosthesis), while the grasping force feedback was provided visually (computer screen). The results indeed showed that the properties of the system and control interface affected the quality of developed internal models and closed-loop control of prosthesis grasping force.

### State Estimation

Sensory information comes from various sources (exteroception, interoception and proprioception) that are characterized by varying level of stochastic noise along with temporal delays. The brain must integrate this information into a composite estimate of our state that also includes knowledge regarding the expected state (internal model estimate).

Optimal estimation incorporates two sources of information by using a weighted average, where the weight assigned to each estimate is a function of its confidence (Ernst and Banks, 2002; Körding and Wolpert, 2004a). For Gaussian distributions, this process is known as Bayesian inference, and humans have been shown across a number of studies to use something similar (see Ernst and Banks, 2002 for seminal work; Kording, 2007 for review). The resulting composite estimate has its own estimate of confidence and may be used to fuse even more sources of information. Therefore, a multitude of sensors may be incorporated into a single estimate of state. A direct consequence of the sensor fusion is that if one signal has substantially more noise than another, incorporating it adds relatively little value, but does not make the net variability worse. This observation is particularly relevant point for supplementary feedback since it is integrated with intrinsic sources, some of which can provide feedback information with high-fidelity (e.g., vision to assess prosthesis motion).

This same concept of data fusion may be applied to the states that are estimated based on internal models. In this case, the final estimate is obtained as a weighted combination of a state estimated from the measurements (sensor data) and that determined by the model. The weighting is known as the Kalman gain, and the process is known as the Kalman filtering. Humans’ state estimation has been well described by Kalman filters (Kording, 2007) and its non-linear extensions (i.e., extended and unscented Kalman filter, particle swarm filter) (Wan and Van Der Merwe, 2001).

The process of state estimation is key to understanding when supplementary sensory feedback in prostheses has worked, and more often, why it has failed. Human subjects can exploit various sources of information to improve motor performance. When somatosensory feedback is missing, as in a deafferented person, the motor control will rely on alternative incidental sensing modalities, such as vision, audition, and vibration (Hermsdörfer et al., 2008). It has been reported a long time ago that amputees can exploit incidental feedback produced by their device (Mann and Reimers, 1970; Prior et al., 1976). In a recent study (Schweisfurth et al., 2019b), it has been shown that visual and auditory cues can be used to estimate prosthesis closing velocity with good precision. Another recent study (Markovic et al., 2018b) demonstrated the ability of subjects to scale prosthesis grasping forces across six different levels from minimum to maximum force by relying only on incidental sources of information (namely, muscle proprioception, vision, and audition). Therefore, contrary to popular thinking, prosthesis control is actually closed-loop even when no explicit somatosensory feedback is transmitted to the prosthesis user.

The contribution of supplementary feedback accordingly depends on its contribution relative to the already-available incidental feedback and the strength of the internal model. As shown in Markovic et al. (2018b), when the supplemental information on the generated force was transmitted through a visual interface after the subjects trained controlling the prosthesis using incidental feedback, the force scaling improved only modestly and mainly at high force levels. It was demonstrated in a recent study (Risso et al., 2019) that an amputee subject with an implanted sensory feedback interface integrated supplementary somatosensory feedback and blurred visual information in a statistically optimal fashion when estimating the size of a hand-held object. If the supplementary feedback is characterized with a higher uncertainty compared to incidental sources, its impact on the control will likely be minimal if any. Therefore, it is critically important that the tactile stimulation profiles used to communicate prosthesis variables through supplementary feedback are easy to discriminate and interpret (Cipriani et al., 2014; Dosen et al., 2017).

### Summary and Implications for Supplementary Feedback in Prostheses

In summary, humans make the best use of the actuators and sensors they have, to achieve the best possible reward they may, considering the probabilistic uncertainty in their control and sensory feedback. Given the structure of these noise sources and the complexity of the tasks humans perform, it as a marvel that they achieve optimal or near-optimal solutions. And yet, this observation offers both perspective and hope as it pertains to prostheses. The best thing going for humans is their brain; not their motors or their sensory receptors. Humans will make use of whatever motors or sensory receptors they have available to achieve the best they can, and in light of the sophisticated control policies they can develop, it is no wonder that many attempts to supplement feedback do not have a significant impact. The brain had already developed an optimal policy that compensated in the control policy for a known deficit in real-time sensory feedback, either through developing internal models, learning to exploit the information from the incidental feedback sources or through navigating control decisions in which sensory feedback was less critical. As we will see below, many studies isolate the role of feedback, but these studies have little explanatory power about the impact of feedback on real-life use of prostheses. In this context, the impact refers not only to the improvement in prosthesis performance (utility), but equally well to enhancing the user experience when interacting with his/her bionic limb by, for example, promoting the feeling of agency and ownership (see section “Psychological aspects”).

Whereas we have noted above and will detail below that the majority of supplementary feedback studies in prostheses focus on real-time feedback, the processes described above require learning and adaptation. This learning and adaptation can only happen in the presence of feedback. Thus, it is quite likely that an equally important role for supplementary feedback is to enable better learning of the task, such that it may be used by internal models and motor control policies. Therefore, it is quite likely that efforts to provide such feedback – particularly in areas where it is not redundantly provided by vision, will have substantial impact on the prosthesis performance (e.g., see Dosen et al., 2015a; Shehata et al., 2018a). The use of feedback in this context can be quite different from its application during real time modulation, e.g., the feedback can be an optional feature that can be activated by the subject when they need to learn the system dynamics. For example, the subject can use feedback during initial practice, and then again, when the system changes the properties due to wear and tear. Nevertheless, this application still needs to be implemented thoughtfully since, as it has been already recognized in the field of motor learning, the feedback can be even detrimental for the learning process if not provided properly (Sigrist et al., 2013).

Given that amputees have the same amazing brain to tackle optimal control problems, but also very different sources of control, mechanism dynamics, and sensory feedback, it will be useful to highlight similarities and differences before moving on to focus on the topic of feedback.

Motor control in able-bodied persons typically starts with the visual observation of the target object. Vision is employed to perceive its extrinsic (position and orientation) and intrinsic (size, shape, and material) properties (MacKenzie and Iberall, 2010). These properties are then used to predict the forces that are needed to grasp and lift the object by employing an inverse model to map the desired outcome (lifting an estimated weight) to the motor commands (muscle excitations and forces) required to achieve that goal (Gordon et al., 1991). After contacting the object, the hand produces forces that are normal and tangential to the object surface, known as grasp and load forces, respectively. The grasp forces establish a firm grip to prevent slippage, while the load forces are responsible for lifting. Importantly, both forces increase simultaneously with the rate of change that is proportional to the estimated object weight, thereby indicating anticipatory control (Johansson and Westling, 1988). If the weight is correctly estimated, this leads to a smooth lifting movement while the object is safely held in the hand. If the estimate is wrong, the subjects can use feedback from a dense network of mechanoreceptors as well as other sources (vision, proprioception) to notice the discrepancy and correct the control (Flanagan et al., 2006).

After an amputation, the sound hand is lost and it is replaced by an artificial system such as a myoelectric prosthesis. The biological connection between the neural controller and its end effector is severed, and replaced by a myoelectric interface, with only incidental feedback from the hand to the user. The prosthesis is controlled by generating myoelectric signals, which are characterized with variability that increases with the contraction intensity (Harris and Wolpert, 1998). The signals are processed and mapped into velocity commands that are sent to the prosthesis, and the resulting motion depends on the mechatronic properties of the system (e.g., communication delays and friction). Current myoelectric prostheses are non-backdrivable systems that are still substantially below the dexterity, precision, and accuracy inherent in biological limbs. A prosthetic device supplies intrinsic feedback to the user. The user can see the prosthesis motion, and in addition, he/she receives mechanical (vibration) and/or auditory (motor and motion sound) cues generated by the moving mechanism. Visual feedback, in particular, can provide high-fidelity information regarding a wide range of modalities (e.g., hand position and grasping force).

**FIGURE 1:**
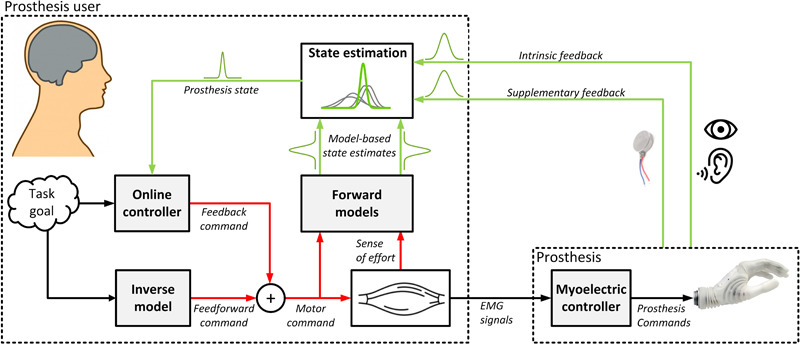
The role of feedback in the larger picture of motor control of a prosthetic arm. The figure illustrates that feedback is only one portion of a broader control paradigm. This should be taken into account when designing methods to provide supplementary feedback. To be successful, the feedback has to make a positive impact within the overall control scheme.

**FIGURE 2:**
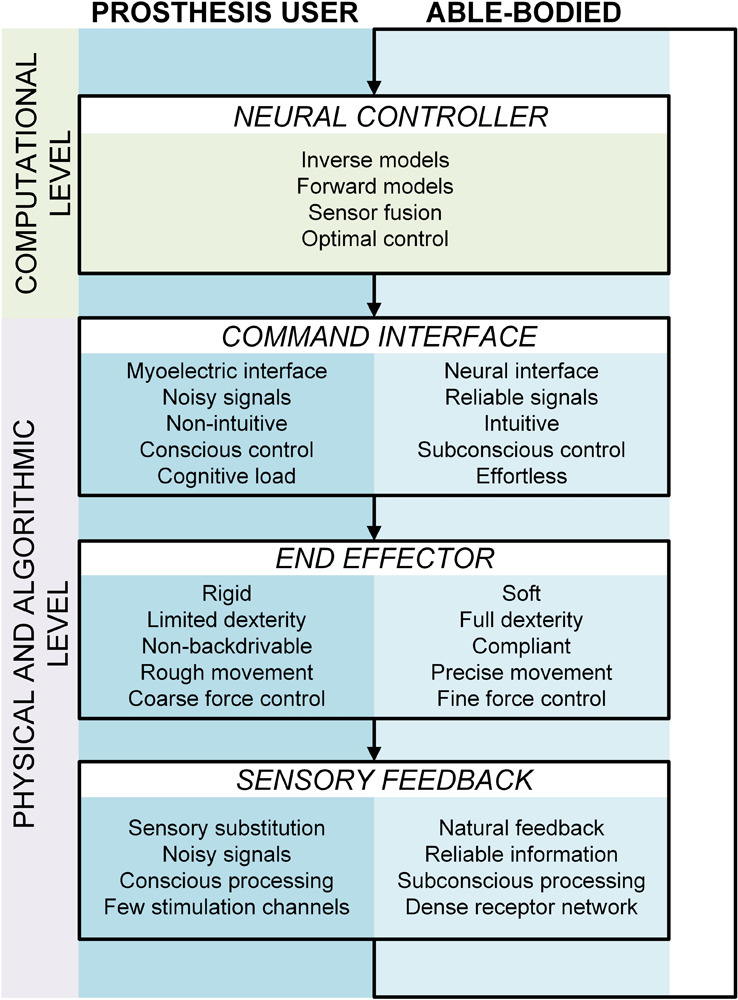
A comparative view of the closed-loop control system in an able-bodied subject (light blue track) versus a prosthesis user (dark blue track). The neural controller (brain) is common to both tracks but the other components (control interface, end effector and sensory feedback) differ fundamentally.

The control loop for using a prosthetic hand includes all the components that are characteristic for the sensory motor control of a sound hand. Figure 1 shows how the artificial extremity integrates into the motor control framework of a prosthesis user. The user relies on internal models to generate feedforward commands directly from the task goal (inverse model) as well as to anticipate the system state (forward model) from the generated control signals (reafference) and interoceptive signals (sense of effort). He/she fuses the model-based prediction with the sensory feedback received from the environment to estimate the state of the prosthesis. This estimate is then used to detect deviations from the task goal, and correct the control if required (online controller). However, there are also crucial differences with respect to the control loop of an able-bodied subject. For example, prosthetic hands are non-backdrivable mechanisms with rough modulation of grasping force. Therefore, a nice and coordinated modulation of the load and grasping forces, characteristic to normal grasping, is not possible. In addition, the lack of precise and reliable control and missing somatosensory feedback affect the ability to acquire as well as update the internal models. Nevertheless, this can change with the development of low-impedance end-effectors (Brown et al., 2015), local feedback loops linearizing the prosthesis behavior (Bottomley, 1965), and with the integration of supplementary feedback into prosthetic systems. Figure 2 highlights the main differences between the components comprising the control loop of an amputee versus an able-bodied subject. Note that the “neural controller” is identical in both cases, emphasizing the assumption that the prosthesis user relies on the same computational mechanisms as an able-bodied subject, but that they must deal with radically different system dynamics and sensory inputs.

## The Role of Feedback for Prosthesis Users

The importance of restoring feedback for prosthesis users is not a new idea. As early as 1917, Rosset (1917) (Patent No. DE301108) had patented a mechanism that relayed finger pressure via pneumatic or mechanical means. Describing his motivation, he said “An artificial limb, especially a hand substitute, will always displease the user because of the missing sensation of touch, when grasping objects. Thus the amputee when using the prosthesis, depends entirely on the visual sense … It is safe to assume that one of the chief reasons arm amputees prefer to do without an artificial hand is the absence of the tactile sense in the substitute.”(Childress, 1980). Work in Italy before 1925 explored similar concepts, mapping finger pressure to thorax skin via pneumatic means (Martin, 1925). Many others followed, including the Vaduz prosthetic hand (Lucaccini et al., 1966) in the 1940’s and patents by Goldman (1951) (Patent No. 2567066) and Gonzelman et al. (1953) (Patent No. US2656545 A). Norbert Weiner, a leader in the field of robotics and prostheses in the mid 20th century said “the present artificial limb removes some of the paralyzes caused by amputation but leaves the ataxia. With the use of proper receptors, much of the ataxia should disappear as well, and the patient should be able to learn reflexes.” (Childress, 1980). It is clear that engineers have been keen to implement feedback solutions throughout the realm of modern prosthesis design.

For classic reviews of feedback, see Childress (1980); Scott (1990), and Kaczmarek et al. (1991). For recent reviews see Schultz and Kuiken (2011); Antfolk et al. (2013c), Schofield et al. (2014), and Svensson et al. (2017). In these manuscripts, the studies investigating supplementary feedback were organized according to the methods used (e.g., invasiveness, stimulation modality). In the present review, on the contrary, we divide the studies based on how and which components of the motor control framework (Figure 1) are impacted by the feedback. Conventional perspectives on feedback in prosthesis control have typically divided feedback into three categories (Childress, 1980). The most popular of these categories—and the focus of this review—is supplementary feedback, i.e., the feedback provided to the user of a prosthesis. Following an extensive review on this topic, the other two categories, which include feedback to change system properties and control-interface feedback, will be briefly summarized (see section “Other applications of feedback”).

### Supplementary Feedback

**FIGURE 3:**
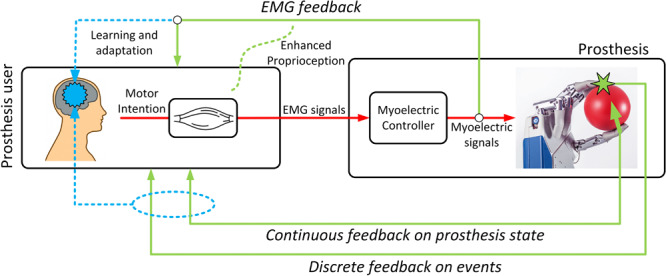
Types of feedback. Feedback is useful for more than real-time continuous regulation. It can supply information about important events (e.g., contact), enhance natural proprioception (sense of effort), and facilitate learning and adaptation through the development of internal models.

The majority of studies have focused on the use of supplementary continuous feedback to improve real-time regulation, as we will see below, but it is important to note that discrete stimulation may also be used, and that the feedback may supply information not only for real-time regulation but also for biofeedback and learning and adaptation. We illustrate these potential impacts of feedback in Figure 3, and review the literature within each one in the following subsections.

#### Continuous Feedback for Real-Time Control

The vast majority of studies have included feedback with a goal of improving real-time closed-loop control (see Antfolk et al., 2013c; Schofield et al., 2014; Svensson et al., 2017 for recent reviews). Childress noted in 1980 that vision was critical as additional source of information (Childress, 1980). Vision supplies information about position and velocity. This information may be used to reliably infer forces – particularly low forces directly after contacting an object (Ninu et al., 2014). Visual cues from deformable objects and the prosthetic hand also supply a surprising amount of context about grasp force. As a result, it is essential when evaluating the clinical utility of any supplementary feedback source to compare it to a baseline of vision. Yet surprisingly, most studies only evaluate supplementary feedback vs. a baseline that occludes vision. In the absence of vision, somatosensory feedback delivered through different interfaces (e.g., vibro-, electro-, and mechanotactile) was shown to be useful in a variety of tasks, such as, controlling hand aperture (Witteveen et al., 2012), grasping force (Witteveen et al., 2015), joint position (Mann and Reimers, 1970; Erwin and Sup, 2015), object size and stiffness discrimination (D’Anna et al., 2019) etc. Of the many studies exploring real-time feedback (see Table A1 in the Appendix), only a few have shown clinical performance improvements in the presence of vision. Each of these will be reviewed below.

Several studies have shown improvement in a virtual reality environment. Although this is a step in the right direction, virtual environments typically do not have the same richness of visual information (for example, virtual objects are completely non-deformable, unlike the real world, where the cosmesis of the prosthetic hand always deforms). Kim and Colgate (2012) showed that providing grasp force via a manual plunger improved performance of a virtual task, using a patient who had targeted sensory reinnervation – a procedure in which afferent fibers that used to go to the hand are rerouted to spare skin (Kuiken et al., 2007a, b; Marasco et al., 2009). Tejeiro et al. (2012) compared mechanical and vibratory feedback of grasp force in a virtual task, and found that both were better than vision alone. Jorgovanovic et al. (2014) used amplitude-modulated electrotactile feedback on grasping force in a virtual prosthesis, and demonstrated that the feedback improved performance while grasping a set of daily life objects of different weights and breaking thresholds. Finally, Dosen et al. (2015a) found that providing visual biofeedback regarding the control signal (processed myoelectric signal) improved control of a virtual hand. In this study, the subjects saw a virtual prosthesis on the computer screen, but they actually controlled a real prosthesis in the background. These studies are each noteworthy in that vision was provided, and yet a convincing improvement was found with supplementary feedback.

There is only one known study in the 20th century that found a clinical improvement using supplementary feedback, namely Meek’s 1989 study (Meek et al., 1989), in which grasp force was conveyed via mechanical means. The subjects were more successful in using prosthesis to grasp and manipulate brittle objects without breaking or dropping them when supplementary feedback was provided in presence of vision compared to vision alone. It is likely that with more subjects and proper statistical analysis, Patterson and Katz would have found similar results in their 1992 study (Patterson and Katz, 1992). Zafar and Van Doren (2000) demonstrated a clinical improvement mapping grasping force to surface electrical stimulation in the presence of vision. Although they used video of a sound hand rather than the device itself, the video was of an actual hand deforming an object, and thus supplied realistic visual cues.

Within the last decade, several groups have made impressive progress along both non-invasive and invasive routes. Gonzalez et al. (2012) has shown that providing hand configuration via audio cues improves performance and reduces mental loading (González et al., 2010). Shehata et al. (2018a) has shown that providing pattern recognition error improves the ability to learn internal models and results in an accompanying improvement in performance. Schweisfurth et al. (2016) showed that myoelectric feedback delivered using electrotactile stimulation with mixed frequency and amplitude coding outperformed conventional force feedback during control of grasping with a prosthetic hand. In a recent study, Markovic et al. (2018a) tested multimodal vibrotactile feedback communicating prosthesis state, contact and force in several functional tasks and across multiple sessions, and demonstrated that the benefits of feedback depended on the task and session (training). Marasco et al. (2018) showed that inducing the kinesthetic illusion in TMR amputees improved real-time feedback (as well as other properties, highlighted below). Cipriani’s group used discrete-event feedback (expanded below), and found an improvement in performance (Clemente et al., 2016; Aboseria et al., 2018). All these studies share a common theme of tapping into a use for feedback that is not redundant with the role played by vision. More specifically, the feedback in these cases transmits variables that are not assessable through vision (e.g., myoelectric signals, change in active function) and/or variables that are difficult to see clearly (e.g., moment of contact with an object), which according to section “State estimation” is likely to improve the overall quality of state estimation.

Regarding invasive techniques, Tan et al. (2014) produced natural electrical feedback in long-term implanted electrodes that conveyed information of finger forces, and demonstrated improved performance of a cherry-picking task. They used specific stimulation properties to mimic natural sensation (Graczyk et al., 2016), and followed up demonstrating improved performance after at-home use (Graczyk et al., 2018). Micera’s group (Valle et al., 2018a) has recently demonstrated similar success with feedback facilitating a delicate task (e.g., virtual egg test).

#### Discrete Feedback for Event Confirmation

As early as 1992, Johansen had developed a paradigm in which the primary role of feedback was to confirm the initiation and termination of discrete events (Johansson and Cole, 1992). Cipriani’s group pursued this idea, developing actuators embedded in electrodes that were able to supply temporally discrete feedback indicating moment of object contact and release. They showed that humans incorporate this feedback, even in the presence of vision, during a grasp and lift task (Cipriani et al., 2014). More recently they have shown that the discrete feedback improves performance (Clemente et al., 2016) and reduces slips (Aboseria et al., 2018). Discrete feedback was largely off the map of prosthetic feedback until the work of Johansen and Cipriani. It is now commercially available and seems likely to have a positive impact on the field. The feedback on contact was also combined with other continuous and discrete modalities, for example, force and velocity (Ninu et al., 2014) and prosthesis state and force (Markovic et al., 2018a). However, in these studies, the individual effects of these modalities on performance were not investigated. A recent study has explored the interaction between discrete tactile feedback and continuous audio biofeedback focusing on the impact that they have on the formation of internal models (Engels et al., 2019). Contrary to expectations, the results seem to imply that when the two modalities were combined, discrete feedback dominated the continuous information. In several studies, the supplementary feedback was used to communicate the event of object slippage prompting the subject to increase the force and prevent losing the object (Aboseria et al., 2018; Zollo et al., 2019).

#### Biofeedback to Facilitate Forward Models (Efference Copy)

Dosen et al. (2015a) study provided feedback regarding the myoelectric signal (Dosen et al., 2015a; Schweisfurth et al., 2016). At first glance, this might seem strange, as it is the user who produced the myoelectric signal in the first place, and furthermore, it is a noisy signal. Why not wait until the signal has produced a movement in the prosthesis, and convey seemingly more useful and less noisy information about prosthesis position, velocity, or force? Our review of computational motor control above suggests two key benefits of providing biofeedback, which has long been used for training and therapeutic motives (Ince et al., 1984). First, supplying feedback at an intermediate stage enables the user to develop more precise internal models of the mechanism – models that are based on the output caused by the actual signal, rather than the intended signal (see Figure 1). This is a noteworthy enhancement. Second, the process of using the myoelectric signal to generate movement takes time, delaying the feedback. Delayed feedback, as we noted above, reduces stable feedback gains. Thus, by relaying the information sooner and allowing the user to predict (using a forward model, or efference copy), they can compensate initially with higher feedback gains, and then correct any minor discrepancies once the final-state feedback arrives using a lower-gain feedback loop (see Figure 1).

#### Delivering Feedback to Improve Feedforward Control (Inverse Model)

Several groups have recently looked at the role of feedback in enabling the development of better internal models. Gillespie et al. (2010) showed that supplementary feedback improved adaptation rates, and internal model development. Saunders and Vijayakumar (2011) demonstrated the importance of inverse models, particularly when control noise was low. Lum et al. (2014) looked at the internal models developed by body-powered prosthesis users, and Johnson et al. (2014, 2017a) looked at the internal models developed by myoelectric prosthesis users. Ninu et al. (2014) showed how vision could reliably convey force information – presumably through an internal model mapping velocity prior to contact to force after contact. Johnson et al. (2017b) manipulated sensory feedback to show its impact on internal model strength. Marasco et al. (2018) demonstrated improved internal model development when kinesthetic illusion was added to targeted muscle reinnervation (TMR) amputees. Shehata et al. (2018a, b, c) demonstrated that improvements in internal model strength via auditory supplementary feedback resulted in improved efficiency and performance. The ability of feedback to improve internal models is a key area to focus in recent and future work.

To promote the use of feedback for the development of internal models, Dosen et al. (2015b) have introduced the paradigm of routine grasping. In this approach to prosthesis control, the subjects are encouraged to close the prosthesis fast by generating feedforward commands. The feedback is therefore not used for online modulation of force as, for example, during slow and careful closing, but for supplying an end-point feedback on the generated force to help adaptation across trials. They have investigated this paradigm and demonstrated (De Nunzio et al., 2017; Strbac et al., 2017) that feedback is useful initially but that its benefits decrease with training, as the subject becomes better in controlling the prosthesis through developed inverse models.

#### Psychological Aspects

Several psychological aspects are influenced by feedback. These aspects are important in their own right, but they also indirectly affect performance. For example, agency has been linked to intentional binding – the subjective binding in time of voluntary actions to their sensory consequences (Haggard et al., 2002; Legaspi and Toyoizumi, 2019), suggesting that when a person has agency over their prosthetic limb, movements seems shorter. It is likely that there is a two-way interaction between the computational motor control, as it applies to a user of a prosthetic limb, and the psychological factors, such as agency, ownership and user experience in general (e.g., improved control leads to better embodiment which might further facilitate the control). Because user dissatisfaction with a lack of agency over their movements has been linked to device abandonment (Biddiss and Chau, 2007; Biddiss et al., 2007), some have suggested that improved agency likely leads to better acceptance of devices (Marasco et al., 2018). These concepts will be briefly reviewed below.

##### Agency

Agency refers to the feeling of controlling actions that influence events in the outside world (Moore and Fletcher, 2012). Some groups have posited that agency arises from processes involved in motor control (Blakemore et al., 2002; Haggard, 2005), and particularly the forward model aspect of internal models (Blakemore et al., 2000, 2002). Other groups believe that agency is formed when external senses are cued (Wegner, 2002, 2003). Recent research has suggested that both motor control and external cues are integral to establishing a sense of agency (Wegner and Sparrow, 2004; Wegner et al., 2004; Synofzik et al., 2008; Moore et al., 2009). In this context agency fits in well with the concept of computational motor control discussed above (Moore and Fletcher, 2012; Legaspi and Toyoizumi, 2019). Within this framework, sensory feedback is critical to both improving the forward models of motor control, affirming motor control via efference copy, and providing relevant contextual feedback that can help with cueing.

Marasco et al. (2018) recently showed that providing kinesthetic feedback via eliciting kinesthetic illusion in targeted muscle reinnervation subjects established a sense of agency over their prosthetic arms. They hypothesized that kinesthesthetic feedback – the sensation of the limb moving in space – was particularly important in creating a sense of agency. It is hopeful that further research by their group and others will further explore the concept of agency.

##### Incorporation

Incorporation is the concept that an object, such as a hand or even a tool such as a hammer, has become part of your body schema. It may be assessed using surveys (Marasco et al., 2018), thermal maps (Marasco et al., 2011), or via temporal judgment assessment tests such as the cross-modal congruency effect (Maravita et al., 2003; Holmes et al., 2004; Blustein et al., 2018b). Providing touch feedback via targeted sensory reinnervation has been shown to improve incorporation (Marasco et al., 2011). The other types of supplementary feedback including vibration, mechanical indentation, and electrical stimulation have demonstrated varying degrees of improved incorporation of a prosthetic limb (Blustein et al., 2018b; Graczyk et al., 2018; Valle et al., 2018a; Cuberovic et al., 2019).

It is noteworthy that whereas recent studies have suggested that dynamic feedback, in the form of kinesthesia, is required to obtain agency, event confirmation feedback, in the form of touch, is required to establish incorporation. Although the topics of agency and incorporation are evolving along with their nomenclature, several researchers have suggested that the combination of incorporation and agency results in embodiment (Longo et al., 2008; Marasco et al., 2018), which is accordingly defined as having agency over your body. It therefore appears that to achieve full embodiment, both kinesthetic and tactile forms of feedback are needed, although further research is required to solidify the possibilities.

##### Phantom Limb Pain

Phantom limb pain is pain perceived as arising from the missing limb due to sources other than stimulation of nociceptive neurons that used to innervate the missing limb (Ortiz-Catalan, 2018). Phantom limb pain can be debilitating and is common after amputation.

It is unclear how phantom limb pain occurs, although there are a number of competing theories including sensory-motor incongruence (similar to motion sickness) (Harris, 1999), cortical reorganization (Flor et al., 1995; Knecht et al., 1998; Lotze et al., 1999, 2001; Grüsser et al., 2001), reduced functional connectivity (Makin et al., 2013), and stochastic entanglement (Ortiz-Catalan, 2018). The latter theory, which is also the most recent one, postulates that stochastic entanglement can occur between networks responsible for sensorimotor processing and paint perception. Many have speculated that phantom limb pain and embodiment are closely connected (Giummarra et al., 2008; Murray, 2008).

Sensory feedback plays a role in all these theories, although not all of them require sensory feedback to alleviate phantom limb pain if motor control is restored.

A number of studies have shown improvements in phantom pain, either through purely therapeutic techniques such as mirror therapy (Chan et al., 2007; Foell et al., 2014) and sensory stimulation/discrimination (Rossini et al., 2010; Horch et al., 2011; Tan et al., 2014), or through actively engaging in the use of the device, as seen through use of myoelectric prostheses (Lotze et al., 1999), targeted muscle reinnervation surgery (Dumanian et al., 2019), or phantom motor execution (Ortiz-Catalan, 2018). Several clinical studies have found that use of devices has reduced phantom limb pain (Lotze et al., 1999; Dumanian et al., 2019), and some laboratory studies have shown reductions in phantom limb pain due to sensory feedback (Rossini et al., 2010; Dietrich et al., 2012, 2018), but no clinical feedback devices are yet available. Based on any of the competing theories, however, it is likely that supplying supplementary sensory feedback would reduce phantom limb pain, and this is a strong area for future research.

### Other Applications of Feedback

Although most studies focus on the use of feedback to provide supplemental information to the user, feedback may also be used to change system properties, and as a type of control interface (Childress, 1980). We briefly review these uses below.

Feedback to change system properties refers to the use of feedback as a part of a local loop within the artificial controller. Many designs within this category use feedback to enable shared control [e.g., artificial reflexes (Salisbury and Colman, 1967; Rakic, 1969; Ring and Welbourn, 1969; Kyberd and Chappell, 1994), computer vision based control (Markovic et al., 2014; Marković et al., 2015; Ghazaei et al., 2017)]. Considering the discussion in the section on agency, when these systems work less than perfectly, relinquishment of autonomy to an external agent might cause frustration by users. Other designs modulate system behavior (e.g., decrease control gain after contact detection; Wettels et al., 2009). These designs enable competing costs such as speed and accuracy to be given prominence during those portions of the task for which they are more likely to be valued, while keeping autonomy with the user. Other designs use feedback to linearize control mechanisms (Bottomley, 1965), which due to static friction, backlash, and resistance from cosmetic gloves are often highly non-linear in prostheses. Although some have commented that this feedback is unnecessary as humans can compensate with visual feedback, the use of feedback to linearize prostheses enables better internal model formation [see Acerbi et al. (2014) for a discussion of difficulties learning more complicated internal models], as well as more reliable control as the local feedback loop can run with less visual delay than the human visual system. Designed properly, these applications of feedback to change system properties can contribute importantly to the closed-loop prosthesis control.

In control-interface feedback, the feedback to the user is inherent in the control process. Driving a powered car is an example of this concept. The control process has been designed in such a way that the user must exert force on the wheel to move it, and if the wheel encounters resistance, this resistance is inherently passed on to the user. Body-powered prostheses provide this form of feedback, as the user can feel the tension in the cable. The Vaduz hand used it as well, routing the force pneumatically (Lucaccini et al., 1966). Simpson termed this concept extended physiological proprioception, and demonstrated its utility across a series of studies in the 1960’s and 1970’s (Simpson, 1972, 1974; Simpson and Smith, 1977). Others have formally quantified the performance of such systems, which combine both control and sensory aspects (Doubler and Childress, 1984a, b). In non-invasive approaches, the end-effector is actuated by moving a body part (e.g., contralateral shoulder) through the cables attached around the body segment, but there is also an invasive version, where the cable of the end effector is connected to the muscle through a skin tunnel created in a surgical operation [i.e., cineplasty (Gale and Hueston, 1957)]. The last extensive research work in this area was done by Weir (1995), and in recent decades the idea has faltered, and is rarely clinically used outside of body-powered prostheses.

## Implications

### Guidelines for Experimental Design/Assessment

**FIGURE 4:**
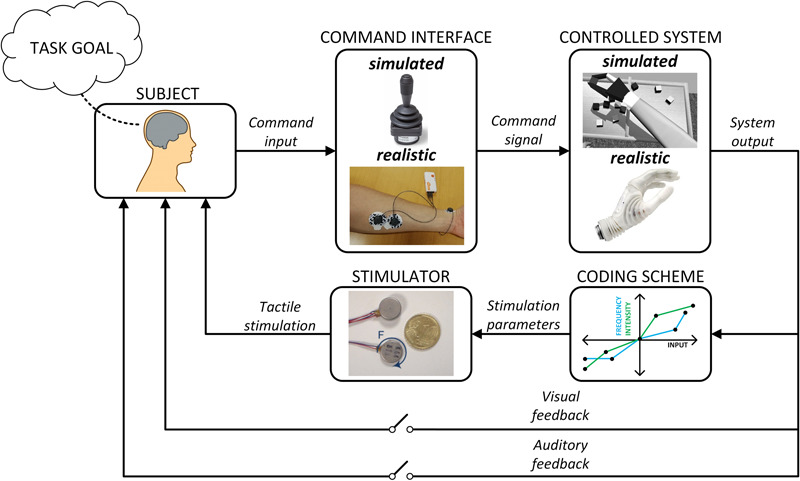
Closed-loop control with supplementary feedback. The interplay between intrinsic feedback sources such as vision and audition with supplementary feedback (including the stimulator and coding scheme) depends on the fidelity of the command interface and the controlled system. These factors can be investigated experimentally by combining virtual and realistic command interfaces and systems, different feedback methods and coding schemes, and allowing or blocking the sources of incidental feedback (e.g., blinded subjects).

A variety of experimental approaches have been used to assess supplemental feedback. Importantly, the methods differ substantially with respect to the level of sensory-motor integration that is embodied by the experimental setup (Figure 4). This in turn determines which components of the motor control loop will be operative in the task, and this is critical in judging the scope of the study outcomes.

The conventional psychometric assessment, which has been used in a number of studies (Szeto and Saunders, 1982; Kaczmarek et al., 1991), investigates sensory experience. In a typical approach, the subject is passive while stimulation is being delivered and he/she is asked to report on the quality and quantity of elicited sensations. In a more interactive setup, the subject can use a joystick to reproduce the intensity and/or frequency of tactile stimulation (e.g., open-loop electrotactile tracking) (Szeto and Lyman, 1977; Anani and Körner, 1979). This allows testing the quality of perception of versatile and dynamic stimulation profiles, but the sensory-motor loop is essentially open.

In closed-loop tracking, the task for the subject is to control a simulated dynamic system using a command interface (e.g., a joystick or myoelectric control) while the feedback on the state of the system is provided through tactile stimulation (Seeley and Bliss, 1966; Schori, 1970; Schmid and Bekey, 1978; Dosen et al., 2014; Paredes et al., 2015). The aim is to generate the control input so that the system output produces a desired reference trajectory. Most commonly, the tactile feedback transmits the momentary tracking error (i.e., so called compensatory tracking; McRuer and Weir, 1969). Therefore, in this experimental paradigm, the subject not only perceives the tactile feedback but also interprets the information and decides on the control action. Compared to simple psychometric testing, this is closer to controlling a real prosthesis. However, some of the components that exist in the realistic control loop (e.g., incidental feedback) are not available in this paradigm. This method has been used to determine the frequency characteristics of the human controller relying on tactile feedback (Schmid and Bekey, 1978), and the impact of stimulation parameters and precision of feedback information (Schori, 1970; Paredes et al., 2015) on the quality of closed-loop control.

Controlling an actual prosthesis while visually and auditory blinding the subjects is a popular approach that is used in many studies in the literature (Raspopovic et al., 2014; Valle et al., 2018b). In reality, this paradigm is not that different from the aforementioned closed-loop tracking, where an actual prosthesis is used in place of a simulated system. Not surprisingly, such experiments consistently demonstrate that the explicit feedback is beneficial for prosthesis control performance. These studies can be used to demonstrate that a particular feedback interface is effective in transmitting desired information, but they do not tell us much about the expected benefits in the actual clinical applications.

In some studies, the subjects can freely observe the prosthesis motion, but the setup is still not fully realistic. For example, the prosthesis can be placed on the table in front of the subject instead of attaching it to the forearm or residual limb (Ninu et al., 2014; Dosen et al., 2015b). The advantage of this approach is that it is possible to investigate specific aspects of the user-prosthesis interaction, while blocking cofounding factors (e.g., prosthesis weight). Finally, the most realistic setup is when the prosthesis is mounted on the subjects and used to accomplish a functional task (Chatterjee et al., 2008; Brown et al., 2015; Pistohl et al., 2015; Clemente et al., 2016, 2019; Raveh et al., 2017; Markovic et al., 2018a).

The motor control perspective discussed in the present manuscript can be used to propose a set of guidelines for designing and conducting experiments evaluating closed-loop prosthesis control. The underlying principle is that the feedback needs to be approached holistically as a component inseparably connected to the other parts of the motor control loop (Figure 1). Therefore, when designing experiments, it is useful to consider, describe and/or address not only the feedback interface but the other segments of the framework as well. This leads us to the following set of recommendations:

• The operation of a prosthesis control interface needs to be clearly explained, so that the level of variability in the generation of control signals can be estimated (or even better, explicitly reported). This variability relates to signal fluctuations around a desired level as well as to the consistency with which different signal levels can be produced across trials.
• The stimulation method and information-coding scheme translating prosthesis variables into stimulation parameters need to be clearly specified and/or psychometrically tested in order to be able to estimate the uncertainty with which the subject can perceive and interpret the feedback. As discussed in section “State estimation,” humans consider both control and sensory noise when developing optimal policies and that is why it is important to describe the characteristics of both noise sources.
• It is important to know the level of experience of a subject participating in the experiment. The experience determines the existence and quality of internal models, and thereby the weight that the subject would place on the feedback versus feedforward approach to control.
• It is relevant to test the proposed closed-loop control interface in subjects with different experience (naïve versus experienced users of myoelectric prostheses) as well as across multiple sessions. The latter is important for assessing the impact of learning and adaptation, and the effect that feedback might have on the development of internal models.
• For studies aiming to demonstrate clinical impact, the performance of developed closed-loop control should be assessed without blocking incidental sources of feedback (e.g., vision and audition) to allow for sensory integration, which will anyway take place during actual clinical use.
• The intended role and application of proposed feedback needs to be clearly stated. For example, is the intention to use the feedback for online modulation or to provide an end-point feedback to facilitate adaptation across trials? Is the feedback aimed at assisting forward and/or inverse model development?

The proposed points are “ideal” requirements and we are fully aware of the challenges that the researchers in this field are facing (e.g., difficulties in recruiting amputee subjects). Therefore, it is clear that it would be very difficult (probably unfeasible) to address all the points within a single study. The aforementioned guidelines should be understood as a list of factors that can be considered and/or discussed to make the study as complete as possible.

### Discussion

In this section, we emphasize certain strategic areas that need to be further investigated in order to design effective interfaces for supplementary feedback in prosthetics. These areas arise directly from the framework that is proposed and discussed in the present manuscript.

Our framework advocates that the challenge of effective closed-loop prosthesis control should be approached from the perspective of human motor control. Therefore, we should first develop a better understanding of how different components (internal models) and motor control processes (estimation, optimal policy) operate in an amputee subject. To this aim, we need theoretical and experimental tools to model, predict and assess those components and processes during myoelectric control and prosthesis operation. We have recently developed methods along this line to measure the strength of internal models in this context (Johnson et al., 2017a; Blustein et al., 2018a; Marasco et al., 2018). In addition, as indicated in sections “Biofeedback to facilitate forward models (efference copy)” and “Delivering feedback to improve feedforward control (inverse model),” some studies have already explicitly addressed the interaction between supplementary feedback and internal models. These tests provided important insights about the interplay between feedforward and feedback mechanisms during prosthesis control as well as some practical guidelines for designing more effective feedback interfaces. The main hypothesis stemming directly from the motor control framework is that to be effective, the feedback needs to be designed so that it makes an impact after it has been integrated with the other components of the motor control loop. Still, almost nothing is known about the cost functions that govern prosthesis control or the optimal policies that amputees use to accomplish different daily life tasks. Shedding light on these components is an imperative to achieve full understanding of optimal control as it applies to prosthetics. This will pave the way for the development of an effective feedback interface, which can make an impact in a daily life of an amputee.

The assessment of feedback is another important topic to be further developed. Presently, it is very difficult to compare the results across different studies since they use substantially different experimental tasks and outcome measures. Most of the clinical tests that are normally used to evaluate prosthesis operation were not really designed to assess the use of closed-loop control. For example, box and blocks, SHAP and clothespin tests can all be accomplished by exerting maximum grasping force, and the grasp economy (e.g., penalizing excessive forces) is not included in the assessment. Therefore, researchers are forced to come up with their own tasks, which leads to a variety of tests. Even in the context of delicate grasping, the selected tasks can be very different, from virtual eggs (Clemente et al., 2016) and sensorized blocks (Meek et al., 1989; Cipriani et al., 2014), which simulate sensitive and brittle objects, to cherry picking (Tyler, 2016) and cup stacking tasks (Markovic et al., 2018a; Clemente et al., 2019) that employ compliant objects. Nevertheless, some of the tests already begin to be applied across research groups (e.g., virtual egg and cup stacking). A promising initiative to develop a standardized battery of tests has been undertaken by the group around HAPTIX project. Importantly, the proposed tests span different scenarios, including an application of Fitts channel capacity to implicit grasp force (Thumser et al., 2018; George et al., 2019), performing functional tasks (e.g., object foraging; Beckler et al., 2019), assessing prosthesis incorporation (Blustein et al., 2018b), and fusing together compensatory motions with eye tracking metrics (Lavoie et al., 2018). A particularly relevant step is the assessment of the prosthesis use longitudinally, across multiple sessions and ideally, in a home environment. And indeed, a recent study has demonstrated that prosthesis performance as well as user experience change dynamically with long-term use (Schofield et al., 2020).

The many methods that are available to provide feedback differ also in the amount of information that they transmit to the subject. Most studies deliver feedback in the form of a continuous tactile signal (e.g., transmitting force through amplitude or frequency of vibrations). Nevertheless, it has been recently proposed to use a low-bandwidth discrete feedback communicating only contact events (Clemente et al., 2016). On the other side, some researchers tested approaches that increase the communication bandwidth, e.g., through the use of visual interfaces [e.g., augmented reality glasses (Clemente et al., 2017; Markovic et al., 2017)] or acoustic signals (Gonzalez et al., 2012; Shehata et al., 2018b). This can be also done through the tactile sense by employing electrodes that integrate a matrix of stimulating pads (Štrbac et al., 2016). Such interfaces can deliver dynamic stimulation patterns that are modulated in location and time and that can communicate multiple feedback variables simultaneously. In addition, matrix electrodes can be used to generate spatially distributed tactile sensations that mimic natural feedback provided by biological hands (e.g., a pressure distribution when grasping an object) (Franceschi et al., 2017; Seminara et al., 2019), especially if coupled with the recent technologies for advanced sensing (e.g., artificial skins; Kim et al., 2014). This research is still in an early stage and it is yet to be investigated what impact such feedback can have on the prosthesis performance and sense of embodiment.

Although outside the scope of this review, the topic of supplementary feedback, particularly seen through the lens of motor control, has important ramifications for our understanding of co-adaptation (e.g., Hahne et al., 2017) and abstract decoding (e.g., Dyson et al., 2018) within the realm of pattern recognition and machine learning. Recent work in this area has benefited from insight within the realm of motor control to provide improved performance (e.g., Ison et al., 2016). As we have argued throughout, the role of feedback is inherently intertwined with that of control and the user (see Figure 1). A specific approach to control can directly affect the intrinsic feedback cues that the user can rely upon when estimating the state. For example, in a conventional proportional controller, the user can estimate the prosthesis grasping force using natural muscle proprioception (sense of contraction) (Markovic et al., 2018b), which is not possible when employing a gated-ramp controller (Humbert et al., 2002; Saunders and Vijayakumar, 2011), where the user can instead rely on the time elapsed from the moment of contact. A better appreciation for these interactions will lead to better feedback, better control, and ultimately, better performance and user satisfaction.

## Conclusion

In summary, supplementary feedback has been investigated for use in prostheses for more than 50 years, but has typically failed to make a clinical impact due to the availability of incidental feedback, the choice of feedback provided, and the inherent noise in many of the sensory feedback information sources. Recent studies have finally started to make a surge in the amount of impactful work in this area. All these works have been designed so that the supplementary feedback makes an impact after integration with the other components of the motor control loop. Many of them have either targeted lower levels of uncertainty (often through invasive techniques), transmitted information that is not already available through the incidental feedback (e.g., myoelectric control signal) or have looked to the role of feedback in providing information outside the realm of real-time control, given that feedback can be an effective instrument for learning and adaptation. As the field continues to advance it is important that we communicate clearly on how each of our studies addresses the various facets of the complicated process (addressed in the guidelines section), and consider the impact of our focused work within the broader process of motor control. Furthermore, this perspective teaches us that feedback and control are essentially inseparable, and therefore, developing prostheses that allow more reliable and sensitive force and position control is an important push towards an effective closed-loop system.

## Author Contributions

Both authors listed have made a substantial, direct and intellectual contribution to the work, and approved it for publication.

## Conflict of Interest

The authors declare that the research was conducted in the absence of any commercial or financial relationships that could be construed as a potential conflict of interest.

## Appendix – List of supplementary feedback studies in prosthesis control

**TABLE A1:** Supplementary sensory feedback in prostheses.

				**Quantitative improvement**		
				**in performance (vision and**		
				**feedback) compared with**		
				**vision alone?**		
		**Experimental**	**Stimulation**	**Virtual**		
**Input**	**Output**	**group**	**position**	**environment**	**Real life**	**References**
Contact pressure	E	TR	RL			Beeker et al., 1967
Grasp force	V	TR	RL			Kawamura and Sueda, 1969
Joint position	V	TH	RL			Mann and Reimers, 1970
Grasp force	N	TR and TH	Median n.			Clippinger et al., 1975
Grasp force	E	TH	RL			Prior and Lyman, 1975
Grasp force, position	N	TR	Radial and Ulnar nerve			Reswick et al., 1975
Grasp force	E	TR	RL			Rohland, 1975
Position, Grasp force	V,E	N.A.	N.A.			Shannon, 1976
Grasp force, position	E	TR	RL			Schmidl, 1973, 1977
Grasp force	E	N.A.	N.A.			Shannon, 1979
Grasp force	E	TR	RL			Kato et al., 1979
Grasp force	E	TR	RL			Scott et al., 1980
Grasp force	D	AB	Forearm		Yes	Meek et al., 1989
Position	E	AB	Array of electrodes in belt			Tupper, 1989
Grasp force	V,D	AB	Upper arm		Yes, but not significant	Patterson and Katz, 1992
Grasp force	E	AB				Wang et al., 1995
Grasp force	E	Nerve injury patients and amputees	Upper arm			Lundborg et al., 1998
Grasp force	E	AB	Neck		Yes	Zafar and Van Doren, 2000
Grasp force, finger position	EPP	Amputees with cineplasty	RL			Weir et al., 2001
Position	N	TR	Median nerve			Dhillon and Horch, 2005
Grasp force	V	TR	RL			Pylatiuk et al., 2006
Touch	D	TSR	Reinnervated area			Kuiken et al., 2007a
Touch	D	TSR	Reinnervated area			Kuiken et al., 2007b
Grasp force	V	AB	Upper arm			Chatterjee et al., 2008
Grasp force	V	AB	Upper arm			Cipriani et al., 2008a
Grasp force	D	AB	Toes			Panarese et al., 2009
Grasp force	D	TSR	Reinnervated area			Sensinger et al., 2009
Vibration	V	TSR	Reinnervated area			Schultz et al., 2009
Orientation, discrimination	D	TSR	Reinnervated area			Marasco et al., 2009
Position	Auditory	AB	Auditory			González et al., 2010
Gripping force	Haptic stylus	AB	Fingers			Stepp and Matsuoka, 2010
Position	Skin stretch	AB	Upper arm			Wheeler et al., 2010
Position and force	N	TR	Ulnar and median n.			Horch et al., 2011
Grasp force	V	AB	Forearm – array			Saunders and Vijayakumar, 2011
Touch	D	TSR	RL			Marasco et al., 2011
Passive hand touch	D	TR	RL			Antfolk et al., 2012
Hand configuration	Auditory	AB	Auditory		Yes	Gonzalez et al., 2012
Grasp force	D	TSR	Reinnervated area	Yes		Kim and Colgate, 2012
Grasp force	V	AB	Upper arm			Stepp and Matsuoka, 2012
Grasp force	V,D	AB	Index finger	Yes		Tejeiro et al., 2012
Pressure	V,D	TR	RL			Antfolk et al., 2013b
Passive hand touch	V,D	TR	RL			Antfolk et al., 2013a
Contact	V	AB	Fingers			Cipriani et al., 2014
Grasp force, velocity	V,D	AB	Forearm			Ninu et al., 2014
Grasp type, aperture size	Augm. reality	AB	Visual			Markovic et al., 2014
Grasping force	E	AB	Forearm	Yes		Jorgovanovic et al., 2014
N.A.	N	TH	Ulnar nerve			Ortiz-Catalan et al., 2014
Grasping force	N	TR	Median, Radial, Ulnar nerves		Yes	Tan et al., 2014
Grasping force	V, joint torque	AB, TR	Forearm			Brown et al., 2015
EMG amplitude	Visual	AB, TR	Visual	Yes		Dosen et al., 2015a
Grasping force	Visual	AB	Visual			Dosen et al., 2015b
Grasp force, position	N	TR	Median, Radial, Ulnar nerves			Schiefer et al., 2016
Contact	V	TR	RL		Yes	Clemente et al., 2016
EMG amplitude	E	AB, TR	Forearm			Schweisfurth et al., 2016
Finger positions	E	AB	Forearm			Patel et al., 2016
Grasp force	V	TR	RL		Yes	Strbac et al., 2017
Grasp force	E	AB	Forearm			Dosen et al., 2017
Grip force, hand aperture	Augm. reality	AB	Visual			Clemente et al., 2017
EMG amplitude, hand aperture, force and contact	Augm. reality, sound	AB	Visual, Audio		Yes	Markovic et al., 2017
Grasp force	V	AB	Forearm			De Nunzio et al., 2017
Grasp force, position	N	TR	Median, Radial, Ulnar nerves			Schiefer et al., 2018
Grasp force, position	N	TR	Median, Radial, Ulnar nerves		Yes	Graczyk et al., 2018
Position	Kinesthetic illusion	TMR	RL		Yes	Marasco et al., 2018
Contact	V	AB	Forearm			Raveh et al., 2017
Pattern rec class velocity	Auditory	AB	Audio		Yes	Shehata et al., 2018a
Contact	V,D	AB	Forearm		Yes	Aboseria et al., 2018
Grasping force	Visual	AB	Visual			Markovic et al., 2018b
Grasping force, state change and contact	V	TR	RL		Yes	Markovic et al., 2018a
Touch, pain	E	TR	RL			Osborn et al., 2018
Grasp force	N	TR	Median, Ulnar		Yes	Valle et al., 2018a
Contact, Grasp force, position	N	TR	Median, Ulnar			George et al., 2019
Grasp force, position	N	TR	Median, Ulnar			Zollo et al., 2019
Grasp force	V, visual	AB	Forearm			Schweisfurth et al., 2019a
Grasp force	E	TR	Ulnar nerve		Yes	Clemente et al., 2019
Grasp force, hand aperture	V	AB	Forearm			Pena et al., 2019
Grasp force, hand aperture	E	TR	Ulnar and median nerves			D’Anna et al., 2019
Hand aperture	Skin stretch	AB, TR	Upper arm			Battaglia et al., 2019
Grasping force	D	TMR	RL			Schofield et al., 2020

*E, electrical surface stimulation; EPP, extended physiological proprioception (combination of sensory feedback and control); N, nerve stimulation; D, direct pressure; V, vibration; AB, able-bodied; TR, transradial amputation; TH, transhumeral amputation; TMR, targeted muscle reinnervation; TSR, targeted sensory reinnervation; RL, residual limb.*

## References

[B1] Aboseria M. Clemente F. Engels L. F. Cipriani C. (2018). Discrete vibro-tactile feedback prevents object slippage in hand Prostheses more intuitively than other modalities. *IEEE Trans. Neural. Syst. Rehabil. Eng.* 26 1577–1584. 10.1109/TNSRE.2018.2851617 29994712

[B2] Acerbi L. Vijayakumar S. Wolpert D. M. (2014). On the origins of suboptimality in human probabilistic inference. *PLoS Compu. Biol.* 10:e1003661. 10.1371/journal.pcbi.1003661 24945142 PMC4063671

[B3] Anani A. B. Körner L. M. (1979). Afferent electrical nerve stimulation: human tracking performance relevant to prosthesis sensory feedback. *Med. Biol. Eng. Comput.* 17 425–434. 10.1007/BF02447053 316053

[B4] Antfolk C. Björkman A. Frank S. Sebelius F. Lundborg G. Rosen B. (2012). Sensory feedback from a prosthetic hand based on air- mediated pressure from the hand to the forearm skin. *J. Rehabil. Med.* 44 702–707. 10.2340/16501977-1001 22729800

[B5] Antfolk C. Cipriani C. Carrozza M. C. Björkman A. Lundborg G. Rosén B. (2013a). Transfer of tactile input from an artificial hand to the forearm: experiments in amputees and able- bodied volunteers. *Disabil. Rehabil.* 8 249–254. 10.3109/17483107.2012.713435 22928878

[B6] Antfolk C. D’Alonzo M. Controzzi M. Lundborg G. Rosen B. Sebelius F. (2013b). Artificial redirection of sensation from prosthetic fingers to the phantom hand map on transradial amputees: vibrotactile versus mechanotactile sensory feedback. *IEEE Trans. Neural. Syst. Rehabil. Eng.* 21 112–120. 10.1109/TNSRE.2012.2217989 23033439

[B7] Antfolk C. D’Alonzo M. Rosén B. Lundborg G. Sebelius F. Cipriani C. (2013c). Sensory feedback in upper limb prosthetics. *Expert Rev. Med. Devices* 10 45–54. 10.1586/erd.12.68 23278223

[B8] Arunachalam A. G. Englehart K. B. Sensinger J. W. (2019). “Optimized control mapping through user-tuned cost of effort, time, and reliability,” in *IEEE International Conference on Rehabilitation Robotics, 2019-June*, Piscataway, NJ: IEEE. 10.1109/ICORR.2019.8779397 31374734

[B9] Battaglia E. Clark J. Bianchi M. Catalano M. Bicchi A. O’Malley M. K. (2019). “Skin stretch haptic feedback to convey closure information in anthropomorphic, under-actuated upper limb soft Prostheses,” in *IEEE Transactions on Haptics*, Piscataway, NJ: IEEE 10.1109/TOH.2019.2915075 31071053

[B10] BBC (1998). *The Man Who Lost His Body.* London: BBC.

[B11] Beckler D. T. Thumser Z. C. Schofield J. S. Marasco P. D. (2019). Using sensory discrimination in a foraging-style task to evaluate human upper-limb sensorimotor performance. *Scie. Rep.* 9:5806. 10.1038/s41598-019-42086-0 30967581 PMC6456599

[B12] Beeker T. During J. de Hertog A. (1967). Artificial touch in a hand prosthesis. *Med. Biol. Eng.* 5 47–49. 10.1007/bf02478841 6037625

[B13] Bernstein N. (1967). *The Co-ordination and Regulation of Movements.* New York, NY: Pergamon Press.

[B14] Bertsekas D. P. (2014). *Dynamic Programming and Optimal Control*, 4th Edn Belmont: Athena Scientific.

[B15] Biddiss E. Beaton D. Chau T. (2007). Consumer design priorities for upper limb prosthetics. *Disabil. Rehabil.* 2 346–357. 10.1080/17483100701714733 19263565

[B16] Biddiss E. A. Chau T. (2007). Upper-limb prosthetics: critical factors in device abandonment. *Am. J. Phys. Med. Rehabil.* 86 977–987. 10.1097/PHM.0b013e3181587f6c 18090439

[B17] Blakemore S. Wolpert D. Frith C. (2002). Abnormalities in the awareness of action. *Trends Cogn. Sci.* 6 237–242. 10.1016/s1364-6613(02)01907-1 12039604

[B18] Blakemore S. J. Wolpert D. Frith C. (2000). Why can’t you tickle yourself? *Neuro Rep.* 11 R11–R16. 10.1097/00001756-200008030-00002 10943682

[B19] Blustein D. Shehata A. Englehart K. Sensinger J. (2018a). Conventional analysis of trial-by-trial adaptation is biased: empirical and theoretical support using a Bayesian estimator. *PloS Comput. Biol.* 14:e1006501. 10.1371/journal.pcbi.1006501 30586387 PMC6324815

[B20] Blustein D. Wilson A. Sensinger J. (2018b). Assessing the quality of supplementary sensory feedback using the crossmodal congruency task. *Sci. Rep.* 8 6203 10.1038/s41598-018-24560-3 PMC5906608 29670188

[B21] Bottomley A. (1965). Myoelectric control of powered Prostheses. *J. Bone Joint Surg. Br.* 47 411–415. 14341052

[B22] Braun D. A. Aertsen A. Wolpert D. M. Mehring C. (2009). Motor task variation induces structural learning. *Curr. Biol.* 19 352–357. 10.1016/j.cub.2009.01.036 19217296 PMC2669412

[B23] Braun D. A. Mehring C. Wolpert D. M. (2010). Structure learning in action. *Behav. Brain Res.* 206 157–165. 10.1016/j.bbr.2009.08.031 19720086 PMC2778795

[B24] Brown J. D. Paek A. Syed M. O’Malley M. K. Shewokis P. A. Contreras-Vidal J. L. (2015). An exploration of grip force regulation with a low-impedance myoelectric prosthesis featuring referred haptic feedback. *J. Neuroeng. Rehabil.* 12:104. 10.1186/s12984-015-0098-1 26602538 PMC4659194

[B25] Chan B. Witt R. Charrow A. Magee A. Howard R. Pasquina P. (2007). Mirror therapy for phantom limb pain. *N. Engl. J. Med.* 357 2206–2207. 18032777 10.1056/NEJMc071927

[B26] Chatterjee A. Chaubey P. Martin J. Thakor N. (2008). Testing a prosthetic haptic feedback simulator with an interactive force matching task. *J. Prosthetics Orthotics* 20 27–34. 10.1097/01.JPO.0000311041.61628.be

[B27] Chhabra M. Jacobs R. A. (2006). Near-optimal human adaptive control across different noise environments. *J. Neurosci.* 26 10883–10887. 10.1523/JNEUROSCI.2238-06.2006 17050726 PMC6674745

[B28] Childress D. S. (1980). Closed-loop control in prosthetic systems - historical perspective. *Ann. Biomed. Eng.* 8 293–303. 10.1007/BF02363433 7027836

[B29] Cipriani C. Segil J. L. Clemente F. Richard R. F. Edin B. (2014). Humans can integrate feedback of discrete events in their sensorimotor control of a robotic hand. *Exp. Brain Res.* 232 3421–3429. 10.1007/s00221-014-4024-8 24992899 PMC4666528

[B30] Cipriani C. Zaccone F. Micera S. Carrozza M. C. (2008). On the shared control of an EMG-controlled prosthetic hand: analysis of user-prosthesis interaction. *IEEE Trans. Rob.* 24 170–184. 10.1109/TRO.2007.910708

[B31] Cisek P. (2009). “Internal Models,” in *Encyclopedia of Neuroscience*, ed. Squire L. R. (Amsterdam: Elsevier).

[B32] Clancy E. A. Bouchard S. Rancourt D. (2001). Estimation and application of EMG amplitude during dynamic contractions. *IEEE Eng. Med. Biol. Mag.* 20 47–54. 10.1109/51.982275 11838258

[B33] Clancy E. A. Morin E. L. Merletti R. (2002). Sampling, noise-reduction and amplitude estimation issues in surface electromyography. *J. Electromyogr. Kinesiol.* 12 1–16. 10.1016/s1050-6411(01)00033-5 11804807

[B34] Clemente F. D’Alonzo M. Controzzi M. Edin B. B. Cipriani C. (2016). Non-invasive, temporally discrete feedback of object contact and release improves grasp control of closed-loop myoelectric transradial Prostheses. *IEEE Trans. Neural. Syst. Rehabil. Eng.* 24 1314–1322. 10.1109/TNSRE.2015.2500586 26584497

[B35] Clemente F. Dosen S. Lonini L. Markovic M. Farina D. Cipriani C. (2017). Humans can integrate augmented reality feedback in their sensorimotor control of a robotic hand. *IEEE Trans. Hum. Mach. Syst.* 47 583–589. 10.1109/THMS.2016.2611998

[B36] Clemente F. Valle G. Controzzi M. Strauss I. Iberite F. Stieglitz T. (2019). Intraneural sensory feedback restores grip force control and motor coordination while using a prosthetic hand. *J. Neural Eng.* 16:026034 10.1088/1741-2552/ab059b 30736030

[B37] Clippinger F. W. Avery R. Titus B. R. (1975). A sensory feedback system for an upper-limb amputation prosthesis. *Bull. Prosthetics Res.* 22 247–258. 4462906

[B38] Cuberovic I. Gill A. Resnik L. J. Tyler D. J. Graczyk E. L. (2019). Learning of artificial sensation through long-term home use of a sensory-enabled prosthesis. *Front. Neurosci.* 13:853 10.3389/fnins.2019.00853 PMC6712074 31496931

[B39] D’Anna E. Valle G. Mazzoni A. Strauss I. Iberite F. Patton J. (2019). A closed-loop hand prosthesis with simultaneous intraneural tactile and position feedback. *Sci. Robo.* 4:eaau8892 10.1101/262741 33137741

[B40] De Luca C. J. (1979). Physiology and mathematics of myoelectric signals. *IEEE Trans. Bio Med. Eng.* 26 313–325. 10.1109/tbme.1979.326534 468280

[B41] De Nunzio A. M. Dosen S. Lemling S. Markovic M. Schweisfurth M. A. Ge N. (2017). Tactile feedback is an effective instrument for the training of grasping with a prosthesis at low- and medium-force levels. *Exp. Brain Res.* 235 2547–2559. 10.1007/s00221-017-4991-7 28550423 PMC5502062

[B42] Dhillon G. S. Horch K. W. (2005). Direct neural sensory feedback and control of a prosthetic arm. *IEEE Trans. Neural. Syst. Rehabil. Eng.* 13 468–472. 10.1109/TNSRE.2005.856072 16425828

[B43] Dietrich C. Nehrdich S. Seifert S. Blume K. R. Miltner W. H. R. Hofmann G. O. (2018). Leg prosthesis with somatosensory feedback reduces phantom limb pain and increases functionality. *Front. Neurol.* 9:270 10.3389/fneur.2018.00270 PMC5932153 29755399

[B44] Dietrich C. Walter-Walsh K. Preissler S. Hofmann G. O. Witte O. W. Miltner W. H. R. (2012). Sensory feedback prosthesis reduces phantom limb pain: proof of a principle. *Neurosci. Lett.* 507 97–100. 10.1016/j.neulet.2011.10.068 22085692

[B45] Dosen S. Markovic M. Somer K. Graimann B. Farina D. (2015a). EMG Biofeedback for online predictive control of grasping force in a myoelectric prosthesis. *J. Neuro Eng. Rehabil.* 12:55 10.1186/s12984-015-0047-z PMC4485858 26088323

[B46] Dosen S. Markovic M. Wille N. Henkel M. Koppe M. Ninu A. (2015b). Building an internal model of a myoelectric prosthesis via closed-loop control for consistent and routine grasping. *Exp. Brain Res.* 233 1855–1865. 10.1007/s00221-015-4257-1 25804864

[B47] Dosen S. Markovic M. Strbac M. Belic M. Kojic V. Bijelic G. (2017). Multichannel electrotactile feedback with spatial and mixed coding for closed-loop control of grasping force in hand Prostheses. *IEEE Trans. Neural. Syst. Rehabil. Eng.* 25 183–195. 10.1109/TNSRE.2016.2550864 27071179

[B48] Dosen S. Schaeffer M.-C. Farina D. (2014). Time-division multiplexing for myoelectric closed-loop control using electrotactile feedback. *J. Neuroeng. Rehabil.* 11:138 10.1186/1743-0003-11-138 PMC4182789 25224266

[B49] Doubler J. A. Childress D. S. (1984a). An analysis of extended physiological proprioception as a prosthesis-control technique. *J. Rehabil. Restorat. Devices* 21 5–18. 6527290

[B50] Doubler J. A. Childress D. S. (1984b). Design and evaluation of a prosthesis control system based on the concept of extended physiological proprioception. *J. Rehabil. Restorat. Devices* 21 19–31. 6527287

[B51] Dumanian G. A. Potter B. K. Mioton L. M. Ko J. H. Cheesborough J. E. Souza J. M. (2019). targeted muscle reinnervation treats neuroma and phantom pain in major limb amputees. *Ann. Surg.* 270 238–246. 10.1097/sla.0000000000003088 30371518

[B52] Dyson M. Barnes J. Nazarpour K. (2018). Myoelectric control with abstract decoders. *J. Neural Eng.* 15:056003 10.1088/1741-2552/aacbfe 29893720

[B53] Engels L. F. Shehata A. W. Scheme E. J. Sensinger J. W. Cipriani C. (2019). When less is more – discrete tactile feedback dominates continuous audio biofeedback in the integrated percept while controlling a myoelectric prosthetic hand. *Front. Neurosci.* 13:578 10.3389/fnins.2019.00578 PMC6563774 31244596

[B54] Ernst M. O. Banks M. (2002). Humans integrate visual and haptic information in a statistically optimal fashion. *Nature* 415:433. 10.1038/415429a 11807554

[B55] Erwin A. Sup F. C. (2015). A haptic feedback scheme to accurately position a virtual wrist prosthesis using a three-node tactor array. *PLoS One* 10:e0134095 10.1371/journal.pone.0134095 PMC4532410 26263015

[B56] Fishbach A. Roy S. A. Bastianen C. Miller L. E. Houk J. C. (2007). Deciding when and how to correct a movement: discrete submovements as a decision making process. *Exp. Brain Res.* 177 45–63. 10.1007/s00221-006-0652-y 16944111

[B57] Fitts P. M. (1954). The information capacity of the human motor system in controlling the amplitude of movement. *J. Exp. Psychol.* 47 381–391. 10.1037/h0055392 13174710

[B58] Flanagan J. R. Bowman M. C. Johansson R. S. (2006). Control strategies in object manipulation tasks. *Curr. Opin. Neurobiol.* 16 650–659. 10.1016/j.conb.2006.10.005 17084619

[B59] Flanagan J. R. King S. Wolpert D. M. Johansson R. S. (2001). Sensorimotor prediction and memory in object manipulation. *Cana. J. Exp. Psychol. Rev. Can. Psychol. Exp.* 55 87–95. 10.1037/h0087355 11433790

[B60] Flanagan J. R. Wing A. M. (1997). The role of internal models in motion planning and control: evidence from grip force adjustments during movements of hand-held loads. *J. Neurosci.* 17 1519–1528. 10.1523/jneurosci.17-04-01519.1997 9006993 PMC6793733

[B61] Flash T. Hogan N. J. (1985). The coordination of arm movements: an experimentally confirmed mathematical model. *J. Neurosci.* 5 1688–1703. 10.1523/jneurosci.05-07-01688.1985 4020415 PMC6565116

[B62] Flor H. Elbert T. Knecht S. Wienbruch C. Pantev C. Birbaumers N. (1995). Phantom-limb pain as a perceptual correlate of cortical reorganization following arm amputation. *Nature* 375 482–484. 10.1038/375482a0 7777055

[B63] Foell J. Bekrater-Bodmann R. Diers M. Flor H. (2014). Mirror therapy for phantom limb pain: brain changes and the role of body representation. *Eur. J. Pain* 18 729–739. 10.1002/j.1532-2149.2013.00433.x 24327313

[B64] Franceschi M. Seminara L. Dosen S. Strbac M. Valle M. Farina D. (2017). A system for electrotactile feedback using electronic skin and flexible matrix electrodes: experimental evaluation. *IEEE Trans. Haptics* 10 162–172. 10.1109/TOH.2016.2618377 27775538

[B65] Gale A. F. Hueston J. T. (1957). Muscle training for biceps cineplasty. *Austr. J. Physiother.* 3 148–151. 10.1016/S0004-9514(14)60934-X

[B66] George J. A. Kluger D. T. Davis T. S. Wendelken S. M. Okorokova E. V. He Q. (2019). Biomimetic sensory feedback through peripheral nerve stimulation improves dexterous use of a bionic hand. *Sci. Rob.* 4:eaax2352 10.1126/scirobotics.aax2352 33137773

[B67] Ghazaei G. Alameer A. Degenaar P. Morgan G. Nazarpour K. (2017). Deep learning-based artificial vision for grasp classification in myoelectric hands. *J. Neural Eng.* 14:036025 10.1088/1741-2552/aa6802 28467317

[B68] Gillespie R. B. Contreras-Vidal J. L. Shewokis P. A. O’Malley M. K. Brown J. D. Davis A. (2010). “Toward improved sensorimotor integration and learning using upper-limb prosthetic devices,” in *2010 Annual International Conference of the IEEE Engineering in Medicine and Biology Society.* Piscataway, NJ: IEEE, 5077–5080. 10.1109/IEMBS.2010.5626206 21096030

[B69] Giummarra M. J. Gibson S. J. Georgiou-Karistianis N. Bradshaw J. L. (2008). Mechanisms underlying embodiment, disembodiment and loss of embodiment. *Neurosci. Biobehav. Rev.* 32 143–160. 10.1016/j.neubiorev.2007.07.001 17707508

[B70] Goldman I. (1951). *Robot Controlled Limb.* U.S. Patent 2,567,066.

[B71] Gonzalez J. Soma H. Sekine M. Yu W. (2012). Psycho-physiological assessment of a prosthetic hand sensory feedback system based on an auditory display: a preliminary study. *J. Neuro Eng. Rehabil.* 9 1–14. 10.1186/1743-0003-9-33 PMC3481360 22682425

[B72] González J. Yu W. Hernandez Arieta A. (2010). Multichannel audio biofeedback for dynamical coupling between prosthetic hands and their users. *Ind. Rob.* 37 148–156. 10.1108/01439911011018920

[B73] Gonzelman J. Ellis H. Clayton O. (1953). *Prosthetic Device Sensory Attachment*. U.S. Patent No. 2656545.

[B74] Gordon A. M. Forssberg H. Johansson R. S. Westling G. (1991). Visual size cues in the programming of manipulative forces during precision grip. *Exp. Brain Res.* 83 477–482. 10.1007/BF00229824 2026190

[B75] Graczyk E. L. Resnik L. Schiefer M. A. Schmitt M. S. Tyler D. J. (2018). Home use of a neural-connected sensory prosthesis provides the functional and psychosocial experience of having a hand again. *Sci. Rep.* 8 1–17. 10.1038/s41598-018-26952-x 29959334 PMC6026118

[B76] Graczyk E. L. Schiefer M. A. Saal H. P. Delhaye B. P. Bensmaia S. J. Tyler D. J. (2016). The neural basis of perceived intensity in natural and artificial touch. *Sci. Trans. Med.* 8 1–11. 10.1126/scitranslmed.aaf5187 PMC5713478 27797958

[B77] Grüsser S. M. Winter C. Muhlnickel W. Denke C. Karl A. Flor H. (2001). The relationship of perceptual phenomena and cortical reorganization in upper extremity amputees. *Neuroscience* 102 263–272. 10.1016/s0306-4522(00)00491-7 11166112

[B78] Guiard Y. Beaudouin-Lafon M. (2004). Fitts’ law 50 years later: applications and contributions from human–computer interaction. *Int. J. Hum. Comput. Stud.* 61 747–750. 10.1016/j.ijhcs.2004.09.003

[B79] Haggard P. (2005). Conscious intention and motor cognition. *Trends Cogn. Sci.* 9 290–295. 10.1016/j.tics.2005.04.012 15925808

[B80] Haggard P. Clark S. Kalogeras J. (2002). Voluntary action and conscious awarenes. *Nat. Neurosci.* 5 382–385. 10.1038/nn827 11896397

[B81] Hahne J. M. Markovic M. Farina D. (2017). User adaptation in myoelectric man-machine interfaces. *Sci. Rep.* 7 1–10. 10.1038/s41598-017-04255-x 28667260 PMC5493618

[B82] Haith A. M. Reppert T. R. Shadmehr R. (2012). Evidence for hyperbolic temporal discounting of reward in control of movements. *J. Neurosci.* 32 11727–11736. 10.1523/JNEUROSCI.0424-12.2012 22915115 PMC3462010

[B83] Harris A. J. (1999). Cortical origin of pathological pain. *Lancet* 354 1464–1466. 10.1016/S0140-6736(99)05003-5 10543687

[B84] Harris C. M. Wolpert D. M. (1998). Signal-dependent noise determines motor planning. *Nature* 394 780–784. 10.1038/29528 9723616

[B85] Hermsdörfer J. Elias Z. Cole J. D. Quaney B. M. Nowak D. A. (2008). Preserved and impaired aspects of feed-forward grip force control after chronic somatosensory deafferentation. *Neurorehabil. Neural Repair.* 22 374–384. 10.1177/1545968307311103 18223241

[B86] Holmes N. P. Calvert G. A. Spence C. (2004). Extending or projecting peripersonal space with tools? Multisensory interactions highlight only the distal and proximal ends of tools. *Neurosci. Lett.* 372 62–67. 10.1016/j.neulet.2004.09.024 15531089

[B87] Horch K. Meek S. Taylor T. G. Hutchinson D. T. (2011). Object discrimination with an artificial hand using electrical stimulation of peripheral tactile and proprioceptive pathways with intrafascicular electrodes. *IEEE Trans. Neural. Syst. Rehabil. Eng.* 19 483–489. 10.1109/TNSRE.2011.2162635 21859607

[B88] Humbert S. D. Snyder S. A. Grill W. M. (2002). Evaluation of command algorithms for control of upper-extremity neural Prostheses. *IEEE Trans. Neural. Syst. Rehabil. Eng.* 10 94–101. 10.1109/TNSRE.2002.1031977 12236452

[B89] Ince L. P. Leon M. S. Christidis D. (1984). Experimental foundations of EMG biofeedback with the upper extremity: a review of the literature. *Biofeedback Self Regul.* 9 371–383. 10.1007/bf00998980 6525360

[B90] Ison M. Vujaklija I. Whitsell B. Farina D. Artemiadis P. (2016). High-density electromyography and motor skill learning for robust long-term control of a 7-DoF robot arm. *IEEE Trans. Neural. Syst. Rehabil. Eng.* 24 424–433. 10.1109/TNSRE.2015.2417775 25838524

[B91] Johansson R. S. Cole K. J. (1992). Sensory-motor coordination during grasping and manipulative actions. *Curr. Opin. Neurobiol.* 2 815–823. 10.1016/0959-4388(92)90139-c 1477545

[B92] Johansson R. S. Westling G. (1988). Coordinated isometric muscle commands adequately and erroneously programmed for the weight during lifting task with precision grip. *Exp. Brain Res.* 71 59–57. 10.1007/BF00247522 3416958

[B93] Johnson R. E. Kording K. P. Hargrove L. J. Sensinger J. W. (2014). Does EMG control lead to distinct motor adaptation? *Front. Neurosci.* 8:302 10.3389/fnins.2014.00302 PMC4179747 25324712

[B94] Johnson R. E. Kording K. P. Hargrove L. J. Sensinger J. W. (2017a). Adaptation to random and systematic errors: comparison of amputee and non-amputee control interfaces with varying levels of process noise. *PLoS One* 12:e0170473 10.1371/journal.pone.0170473 PMC5354256 28301512

[B95] Johnson R. E. Kording K. P. Hargrove L. J. Sensinger J. W. (2017b). EMG versus torque control of human-machine systems: equalizing control signal variability does not equalize error or uncertainty. *IEEE Trans. Neural. Syst. Rehabil. Eng.* 25 660–667. 10.1109/TNSRE.2016.2598095 27576255

[B96] Jones K. E. Hamilton A. F. Wolpert D. M. (2002). Sources of signal-dependent noise during isometric force production. *J. Neurophysiol.* 88 1533–1544. 10.1152/jn.00985.2001 12205173

[B97] Jorgovanovic N. Dosen S. Djozic D. J. Krajoski G. Farina D. (2014). Virtual grasping: closed-loop force control using electrotactile feedback. *Comput. Math. Methods Med.* 2014:120357 10.1155/2014/120357 PMC3909980 24516504

[B98] Kaczmarek K. A. Webster J. G. Bach-y-rita P. Tompkins W. J. (1991). Electrotactile and vibrotactile displays for sensory substition systems. *IEEE Trans. Biomed. Eng.* 38 1–16. 10.1109/10.68204 2026426

[B99] Kato I. Yamakawa S. Ichikawa K. Sano M. (1979). “Multifunctional myoelectric hand prosthesis with pressure sensory feedback system: Waseda hand,” in *Advances in External Control of Human Extremities ETAN*, Dubrovnik, 155–170.

[B100] Kawamura Z. Sueda O. (1969). “Sensory feedback device for the artificial arm,” in *4th Pan Pacific Rehabilitation Conference*, Osaka.

[B101] Kawato M. (1999). Internal models for motor control and trajectory planning. *Curr. Opin. Neurobiol.* 9 718–727. 10.1016/S0959-4388(99)00028-8 10607637

[B102] Kim J. Lee M. Shim H. J. Ghaffari R. Cho H. R. Son D. (2014). Stretchable silicon nanoribbon electronics for skin prosthesis. *Nat, Commun.* 5:5747 10.1038/ncomms6747 25490072

[B103] Kim K. Colgate J. E. (2012). Haptic feedback enhances grip force control of sEMG-controlled prosthetic hands in targeted reinnervation amputees. *IEEE Trans. Neural. Syst. Rehabil. Eng.* 20 798–805. 10.1109/TNSRE.2012.2206080 22855230

[B104] Knecht S. Henningsen H. Hohling C. Elbert T. Flor H. Pantev C. (1998). Plasticity of plasticity? Changes in the pattern of perceptual correlates of reorganization after amputation\rPhantom-limb pain as a perceptual correlate of cortical reorganization following arm amputation. *Brain* 121(Pt 4), 717–724. 10.1093/brain/121.4.717 9577396

[B105] Kording K. (2007). Decision theory: what “should” the nervous system do? *Science* 318 606–610. 10.1126/science.1142998 17962554

[B106] Körding K. P. Wolpert D. M. (2004a). Bayesian integration in sensorimotor learning. *Nature* 427 244–247. 10.1038/nature02169 14724638

[B107] Körding K. P. Wolpert D. M. (2004b). The loss function of sensorimotor learning. *Proc. Natl. Acad. Sci. U.S.A.* 101 9839–9842. 10.1073/pnas.0308394101 15210973 PMC470761

[B108] Kositsky M. Barto A. G. (2001). The emergence of multiple movement units in the presence of noise and feedback delay. *Adv. Neural Inform. Process. Syst.* 44-46 889–895. 10.1016/s0925-2312(02)00488-5

[B109] Kuiken T. A. Marasco P. D. Lock B. A. Harden R. N. Dewald J. P. A. (2007a). Redirection of cutaneous sensation from the hand to the chest skin of human amputees with targeted reinnervation. *Proc. Natl. Acad. Sci. U.S.A.* 104 20061–20066. 10.1073/pnas.0706525104 18048339 PMC2148422

[B110] Kuiken T. A. Miller L. A. Lipschutz R. D. Lock B. A. Stubblefield K. Marasco P. D. (2007b). Targeted reinnervation for enhanced prosthetic arm function in a woman with a proximal amputation: a case study. *Lancet* 369 371–380. 10.1016/S0140-6736(07)60193-7 17276777

[B111] Kyberd P. J. Chappell P. H. (1994). The Southampton hand - an intelligent myoelectric prosthesis. *J. Rehabil. Res. Dev.* 31 326–334. 7869280

[B112] Lambrecht J. M. Pulliam C. L. Kirsch R. F. (2011). Virtual reality environment for simulating tasks with a myoelectric prosthesis: an assessment and training tool. *J. Prosthet. Orthot.* 23 89–94. 10.1097/jpo.0b013e318217a30c 23476108 PMC3589581

[B113] Lavoie E. B. Valevicius A. M. Boser Q. A. Kovic O. Vette A. H. Pilarski P. M. (2018). Using synchronized eye and motion tracking to determine high-precision eye-movement patterns during objectinteraction tasks. *J. Vis.* 18 1–20. 10.1167/18.6.18 30029228

[B114] Legaspi R. Toyoizumi T. (2019). A bayesian psychophysics model of sense of agency. *Nat. Commun.* 10 1–11. 10.1038/s41467-019-12170-0 31534122 PMC6751175

[B115] Li W. Todorov E. (2007). Iterative linearization methods for approximately optimal control and estimation of non-linear stochastic system. *Int. J. Control* 80 1439–1453. 10.1080/00207170701364913

[B116] Liu D. Todorov E. (2007). Evidence for the flexible sensorimotor strategies predicted by optimal feedback control. *J. Neurosci.* 27 9354–9368. 10.1523/JNEUROSCI.1110-06.2007 17728449 PMC6673117

[B117] Longo M. R. Schüür F. Kammers M. P. M. Tsakiris M. Haggard P. (2008). What is embodiment? A psychometric approach. *Cognition* 107 978–998. 10.1016/j.cognition.2007.12.004 18262508

[B118] Lotze M. Flor H. Grodd W. Larbig W. Birbaumer N. (2001). Phantom movements and pain. An fMRI study in upper limb amputees. *Brain A J. Neurol.* 124(Pt 11), 2268–2277. 10.1093/brain/124.11.2268 11673327

[B119] Lotze M. Grodd W. Birbaumer N. Erb M. Huse E. Flor H. (1999). Does use of a myoelectric prosthesis prevent cortical reorganization and phantom limb pain? *Nat. Neurosci.* 2 501–502. 10.1038/9145 10448212

[B120] Lucaccini L. Kaiser P. Lyman J. (1966). The French electric hand: some observations and conclusions. *Bull. Prosthetics Res.* 30–51.

[B121] Lum P. S. Black I. Holley R. J. Barth J. Dromerick A. W. (2014). Internal models of upper limb prosthesis users when grasping and lifting a fragile object with their prosthetic limb. *Exp. Brain Res.* 232 3785–3795. 10.1007/s00221-014-4071-1 25142151

[B122] Lundborg G. Rosén B. Lindström K. Lindberg S. (1998). Artificial sensibility based on the use of piezoresistive sensors. Preliminary observations. *J. Hand Surg.* 23 620–626. 10.1016/s0266-7681(98)80016-8 9821608

[B123] MacKenzie C. Iberall T. (2010). *The Grasping Hand.* Amsterdam: Elsevier.

[B124] Makin T. R. Scholz J. Filippini N. Henderson Slater D. Tracey I. Johansen-Berg H. (2013). Phantom pain is associated with preserved structure and function in the former hand area. *Nat. Commun.* 4 1570–1578. 10.1038/ncomms2571 23463013 PMC3615341

[B125] Mann R. Reimers S. (1970). Kinesthetic sensing for the EMG controlled Boston arm. *IEEE Trans. Man Mach. Syst.* 11 110–115. 10.1109/tmms.1970.299971

[B126] Marasco P. D. Hebert J. S. Sensinger J. W. Shell C. E. Schofield J. S. Orzell B. M. (2018). Illusory movement perception improves motor control for prosthetic hands. *Sci. Trans. Med.* 10 1–13. 10.1126/scitranslmed.aao6990 PMC5906050 29540617

[B127] Marasco P. D. Kim K. Colgate J. E. Peshkin M. A. Kuiken T. A. (2011). Robotic touch shifts perception of embodiment to a prosthesis in targeted reinnervation amputees. *Brain* 134(Pt 3), 747–758. 10.1093/Brain/Awq361 21252109 PMC3044830

[B128] Marasco P. D. Schultz A. E. Kuiken T. A. (2009). Sensory capacity of reinnervated skin after redirection of amputated upper limb nerves to the chest. *Brain* 132(Pt 6), 1441–1448. 10.1093/brain/awp082 19369486 PMC2685921

[B129] Maravita A. Spence C. Driver J. (2003). Multisensory integration and the body schema: close to hand and within reach. *Curr. Biol.* 13 R531–R539. 10.1016/S0960-9822(03)00449-4 12842033

[B130] Markovic M. Dosen S. Cipriani C. Popovic D. Farina D. (2014). Stereovision and augmented reality for closed-loop control of grasping in hand Prostheses. *J. Neural Eng.* 11:046001 10.1088/1741-2560/11/4/046001 24891493

[B131] Marković M. Dosen S. Popović D. B. Graimann B. Farina D. (2015). Computer vision and sensor fusion for semi-autonomous control of a multi degree-of-freedom prosthesis. *J. Neural Eng.* 12:066022 10.1088/1741-2560/12/6/066022 26529274

[B132] Markovic M. Karnal H. Graimann B. Farina D. Dosen S. (2017). GLIMPSE: google Glass interface for sensory feedback in myoelectric hand Prostheses. *J. Neural Eng.* 14:036007 10.1088/1741-2552/aa620a 28355147

[B133] Markovic M. Schweisfurth M. A. Engels L. F. Bentz T. Wüstefeld D. Farina D. (2018a). The clinical relevance of advanced artificial feedback in the control of a multi-functional myoelectric prosthesis. *J. Neuroeng. Rehabil.* 15:28 10.1186/s12984-018-0371-1 PMC5870217 29580245

[B134] Markovic M. Schweisfurth M. A. Engels L. F. Farina D. Dosen S. (2018b). Myocontrol is closed-loop control: incidental feedback is sufficient for scaling the prosthesis force in routine grasping. *J. Neuro Eng. Rehabil.* 15:81 10.1186/s12984-018-0422-7 PMC6122439 30176929

[B135] Marr D. (1982). *A Computational Investigation into the Human Representation and Processing of Visual Information.* New York, NY: Henry Holt and Co.

[B136] Martin F. (1925). *Artificial Limbs.* Geneva: International Labour Office, Studies and Reports, Series No. 5.

[B137] McRuer D. Weir D. (1969). Theory of manual vehicular control. *IEEE Trans. Man Mach. Syst.* 10 257–291. 10.1109/TMMS.1969.299930 5823971

[B138] Meek S. G. Jacobsen S. C. Goulding P. (1989). Extended physiologic taction: design and evaluation of a proportional force feedback system. *J. Rehabil. Res. Dev.* 26 53–62. 2666644

[B139] Micera S. Carpaneto J. Raspopovic S. (2010). Control of hand Prostheses using peripheral information. *IEEE Rev. Biomed. Eng.* 3 48–68. 10.1109/rbme.2010.2085429 22275201

[B140] Mitrovic D. Klanke S. Osu R. Kawato M. Vijayakumar S. (2010). A computational model of limb impedance control based on principles of internal model uncertainty. *PLoS One* 5:e13601 10.1371/journal.pone.0013601 PMC2964289 21049061

[B141] Moore J. Wegner D. Haggard P. (2009). Modulating the sense of agency with external cues. *Conscious Cogn.* 18 1056–1064. 10.1016/j.concog.2009.05.004 19515577

[B142] Moore J. W. Fletcher P. C. (2012). Sense of agency in health and disease: a review of cue integration approaches. *Conscious Cogn.* 21 59–68. 10.1016/j.concog.2011.08.010 21920777 PMC3315009

[B143] Murray C. D. (2008). “Embodiment and Prosthetics,” in *Psychoprosthetics: State of the Knowledge*, eds Gallagher P. Desmond D. M. MacLachlan M. (Berlin: Springer).

[B144] Nagengast A. J. Braun D. A. Wolpert D. M. (2010). Risk-sensitive optimal feedback control accounts for sensorimotor behavior under uncertainty. *PLoS Comput. Biol.* 6:e1000857 10.1371/journal.pcbi.1000857 PMC2904762 20657657

[B145] Ninu A. Dosen S. Muceli S. Rattay F. Dietl H. Farina D. (2014). Closed-loop control of grasping with a myoelectric hand prosthesis: which are the relevant feedback variables for force control? *IEEE Trans. Neural Syst. Rehabil. Eng.* 22 1041–1052. 10.1109/TNSRE.2014.2318431 24801625

[B146] Ortiz-Catalan M. (2018). The stochastic entanglement and phantom motor execution hypotheses: a theoretical framework for the origin and treatment of Phantom limb pain. *Front. Neurol.* 9:748 10.3389/fneur.2018.00748 PMC6135916 30237784

[B147] Ortiz-Catalan M. Håkansson B. Brånemark R. (2014). An osseointegrated human-machine gateway for long-term sensory feedback and motor control of artificial limbs. *Sci. Trans. Med.* 6:257re6 10.1126/scitranslmed.3008933 25298322

[B148] Osborn L. E. Dragomir A. Betthauser J. L. Hunt C. L. Nguyen H. H. Kaliki R. R. (2018). Prosthesis with neuromorphic multilayered e-dermis perceives touch and pain. *Sci. Rob.* 3:eaat3818 10.1126/scirobotics.aat3818 PMC7051004 32123782

[B149] Osu R. Burdet E. Franklin D. W. Milner T. E. Kawato M. (2003). Different mechanisms involved in adaptation to stable and unstable dynamics. *J. Neurophysiol.* 90 3255–3269. 10.1152/jn.00073.2003 14615431

[B150] O’Sullivan I. Burdet E. Diedrichsen J. (2009). Dissociating variability and effort as determinants of coordination. *PLoS Comput. Biol.* 5:e1000345 10.1371/journal.pcbi.1000345 PMC2661023 19360132

[B151] Panarese A. Edin B. B. Vecchi F. Carrozza M. C. Johansson R. S. Member A. (2009). Humans can integrate force feedback to toes in their sensorimotor control of a robotic hand. *IEEE Trans. Neural. Syst. Rehabil. Eng.* 17 560–567. 10.1109/TNSRE.2009.2021689 19457753

[B152] Paredes L. P. Dosen S. Rattay F. Graimann B. Farina D. (2015). The impact of the stimulation frequency on closed-loop control with electrotactile feedback. *J. Neuro Eng. Rehabil.* 12:35 10.1186/s12984-015-0022-8 PMC4403675 25889752

[B153] Pasluosta C. Kiele P. Stieglitz T. (2018). Paradigms for restoration of somatosensory feedback via stimulation of the peripheral nervous system. *Clin. Neurophysiol.* 129 851–862. 10.1016/j.clinph.2017.12.027 29343415

[B154] Patel G. K. Dosen S. Castellini C. Farina D. (2016). Multichannel electrotactile feedback for simultaneous and proportional myoelectric control. *J. Neural Eng.* 13:056015 10.1088/1741-2560/13/5/056015 27618968

[B155] Patterson P. E. Katz J. A. (1992). Design and evaluation of a sensory feedback-system that provides grasping pressure in a myoelectric hand. *Bull. Prosthetics Res.* 29 1–8. 10.1682/jrrd.1992.01.0001 1740774

[B156] Pena A. E. Rincon-Gonzalez L. Abbas J. J. Jung R. (2019). Effects of vibrotactile feedback and grasp interface compliance on perception and control of a sensorized myoelectric hand. *PLoS One* 14:e0210956 10.1371/journal.pone.0210956 PMC6334959 30650161

[B157] Pistohl T. Joshi D. Ganesh G. Jackson A. Nazarpour K. (2015). Artificial proprioceptive feedback for myoelectric control. *IEEE Trans. Neural. Syst. Rehabil. Eng.* 23 498–507. 10.1109/TNSRE.2014.2355856 25216484 PMC7610977

[B158] Prior R. Lyman J. (1975). Electrocutaneous feedback for artificial limbs. *Bull. Prosthetics Res.* 10 3–37. 1227686

[B159] Prior R. E. Lyman J. Case P. A. Scott C. M. (1976). Supplemental sensory feedback for the VA/NU myoelectric hand. Background and preliminary designs. *Bull. Prosthetics Res.* 10–26 170–191. 1030327

[B160] Pylatiuk C. Kargov A. Schulz S. (2006). Design and evaluation of a low-cost force feedback system for myoelectric prosthetic hands. *J. Prosthetics Orthotics* 18 57–61. 10.1097/00008526-200604000-00007

[B161] Rakic M. (1969). The Belgrade hand prosthesis. *Proc. Inst. Mech. Eng.* 183 60–67. 10.1243/pime_conf_1968_183_179_02

[B162] Raspopovic S. Capogrosso M. Petrini F. M. Bonizzato M. Rigosa J. Micera S. (2014). Restoring natural sensory feedback in real-time bidirectional hand Prostheses. *Sci. Trans. Med.* 6:222ra19 10.1126/scitranslmed.3006820 24500407

[B163] Raveh E. Friedman J. Portnoy S. (2017). Visuomotor behaviors and performance in a dual-task paradigm with and without vibrotactile feedback when using a myoelectric controlled hand. *Assist. Technol.* 30 274–280. 10.1080/10400435.2017.1323809 28628379

[B164] Reinkensmeyer D. J. Guigon E. Maier M. A. (2012). A computational model of use-dependent motor recovery following a stroke: optimizing corticospinal activations via reinforcement learning can explain residual capacity and other strength recovery dynamics. *Neural Networks* 2 60–69. 10.1016/j.neunet.2012.02.002 PMC3678524 22391058

[B165] Reswick J. Mooney V. Schwartz A. McNeal D. Su N. Sperry C. (1975). “Sensory feedback prosthesis using intraneural electrodes,” in *Proceedings of the 5th International Symposium on External Control of Human Extremities*, Dubrovnik, 9–24.

[B166] Rigoux L. Guigon E. (2012). A model of reward- and effort-based optimal decision making and motor control. *PLoS Comput. Biol.* 8:e1002716 10.1371/journal.pcbi.1002716 PMC3464194 23055916

[B167] Ring N. Welbourn D. (1969). A self-adaptive gripping device: its design and performance. *Proc. Inst. Mech. Eng.* 183 45–49. 10.1243/pime_conf_1968_183_176_02

[B168] Risso G. Valle G. Iberite F. Strauss I. Stieglitz T. Controzzi M. (2019). Optimal integration of intraneural somatosensory feedback with visual information: a single-case study. *Scie. Rep.* 9:7916 10.1038/s41598-019-43815-1 PMC6536542 31133637

[B169] Rohland T. (1975). Sensory feedback for powered limb Prostheses. *Med. Biol. Eng.* 13 300–301. 10.1007/bf02477743 1195823

[B170] Rosset F. (1917). *Patent No. DE301108. Germany.*

[B171] Rossini P. M. Micera S. Benvenuto A. Carpaneto J. Cavallo G. Citi L. (2010). Double nerve intraneural interface implant on a human amputee for robotic hand control. *Clin. Neurophysiol.* 121 777–783. 10.1016/j.clinph.2010.01.001 20110193

[B172] Salisbury L. Colman A. (1967). A mechanical hand with automatic proportional control of prehension. *Med. Biol. Eng.* 5 505–511. 10.1007/bf02479145 6056363

[B173] Saunders I. Vijayakumar S. (2011). The role of feed-forward and feedback processes for closed-loop prosthesis control. *J. Neuroeng. Rehabil.* 8:60 10.1186/1743-0003-8-60 PMC3227590 22032545

[B174] Schiefer M. Tan D. Sidek S. M. Tyler D. J. (2016). Sensory feedback by peripheral nerve stimulation improves task performance in individuals with upper limb loss using a myoelectric prosthesis. *J. Neural Eng.* 13:016001 10.1088/1741-2560/13/1/016001 PMC5517302 26643802

[B175] Schiefer M. A. Graczyk E. L. Sidik S. M. Tan D. W. Tyler D. J. (2018). Artificial tactile and proprioceptive feedback improves performance and confidence on object identification tasks. *PLoS One* 13:e0207659 10.1371/journal.pone.0207659 PMC6281191 30517154

[B176] Schmid H. P. Bekey G. A. (1978). Tactile information processing by human operators in control systems. *IEEE Trans. Syst.ManCybern.* 8 860–866. 10.1109/TSMC.1978.4309886

[B177] Schmidl H. (1973). The INAIL-CECA Prostheses. *Orthotics Prosthetics* 27 6–12.

[B178] Schmidl H. (1977). The importance of information feedback in Prostheses for the upper limbs. *Prosthetics Orthotics Int.* 1 21–24. 10.3109/03093647709164601 615983

[B179] Schofield J. S. Evans K. R. Carey J. P. Hebert J. S. (2014). Applications of sensory feedback in motorized upper extremity prosthesis: a review. *Expert Rev. Med. Devices* 11 499–511. 10.1586/17434440.2014.929496 24928327

[B180] Schofield J. S. Shell C. E. Beckler D. T. Thumser Z. C. Marasco P. D. (2020). Long-term home-use of sensory-motor-integrated bidirectional bionic prosthetic arms promotes functional, perceptual, and cognitive changes. *Front. Neurosci.* 14:120 10.3389/fnins.2020.00120 PMC7042391 32140096

[B181] Schori T. R. (1970). Tracking performance as a function of precision of electrocutaneous feedback information. *Hum. Fact.* 12 447–452. 10.1177/001872087001200503

[B182] Schrater P. Kording K. P. Blohm G. (2019). “Modeling in neuroscience as a decision process,” in *Conference on Cognitive Computational Neuroscience*, San Francisco, CA.

[B183] Schultz A. E. Kuiken T. A. (2011). Neural interfaces for control of upper limb Prostheses: the state of the art and future possibilities. *J. Inj. Funct.Rehabil.* 3 55–67. 10.1016/j.pmrj.2010.06.016 21257135

[B184] Schultz A. E. Marasco P. D. Kuiken T. A. (2009). Vibrotactile detection thresholds for chest skin of amputees following targeted reinnervation surgery. *Brain Res.* 1251 121–129. 10.1016/j.brainres.2008.11.039 19059226

[B185] Schweisfurth M. A. Hartmann C. Schimpf F. Farina D. Dosen S. (2019a). “The interaction between feedback type and learning in routine grasping with myoelectric Prostheses,” in *IEEE Transactions on Haptics*, Piscataway, NJ. 10.1109/TOH.2019.2961652 31870991

[B186] Schweisfurth M. A. Niethammer C. Meyer B. Farina D. Dosen S. (2019b). Psychometric characterization of incidental feedback sources during grasping with a hand prosthesis. *J Neuro Eng. Rehabil.* 16:155. 10.1186/s12984-019-0622-9 PMC6902515 31823792

[B187] Schweisfurth M. A. Markovic M. Dosen S. Teich F. Graimann B. Farina D. (2016). Electrotactile EMG feedback improves the control of prosthesis grasping force. *J. Neural Eng.* 13:056010 10.1088/1741-2560/13/5/056010 27547992

[B188] Scott R. N. (1990). Feedback in myoelectric Prostheses. *Clin. Orthop.* 256 58–63. 2194730

[B189] Scott R. N. Brittain R. H. Caldwell R. R. Cameron A. B. Dunfield V. A. (1980). Sensory-feedback system compatible with myoelectric control. *Med. Biol. Eng. Compu.* 18 65–69. 10.1007/bf02442481 7382591

[B190] Scott S. H. (2004). Optimal feedback control and the neural basis of volitional motor control. *Nat. Rev. Neurosci.* 5 532–546. 10.1038/nrn1427 15208695

[B191] Seeley H. F. Bliss J. C. (1966). Compensatory tracking with visual and tactile displays. *IEEE Trans. Hum. Fact. Electron.* 7 84–90. 10.1109/THFE.1966.232328

[B192] Seminara L. Fares H. Franceschi M. Valle M. Strbac M. Farina D. (2019). Dual-parameter modulation improves stimulus localization in multichannel electrotactile stimulation. *IEEE Trans. Haptics.* 10.1109/TOH.2019.2950625 [Epub ahead of print]. 31675343

[B193] Sensinger J. W. Aleman-Zapata A. Englehart K. (2015). Do cost functions for tracking error generalize across tasks with different noise levels? *PloS one* 10:e0136251 10.1371/journal.pone.0136251 PMC4552421 26313560

[B194] Sensinger J. W. Schultz A. E. Kuiken T. A. (2009). Examination of force discrimination in human upper limb amputees with reinnervated limb sensation following peripheral nerve transfer. *IEEE Trans. Neural. Syst. Rehabil. Eng.* 17 438–444. 10.1109/TNSRE.2009.2032640 19775983 PMC3025706

[B195] Sensinger J. W. Hill W. Sybring M. (2019). “Prostheses-Assistive Technology-Upper,” in *Encyclopedia of Biomedical Engineering*, Editor in Chief Narayan R. eds Wang M. Laurencin C. Yu X. (Amsterdam: Elsevier), 632–644. 10.1016/b978-0-12-801238-3.99912-4

[B196] Shadmehr R. Krakauer J. W. (2008). A computational neuroanatomy for motor control. *Exp. Brain Res.* 185 359–381. 10.1007/s00221-008-1280-5 18251019 PMC2553854

[B197] Shadmehr R. Mussa-Ivaldi F. A. (1994). Adaptive representation of dynamics during learning of a motor task. *J. Neurosci.* 74 3208–3224. 10.1523/jneurosci.14-05-03208.1994 PMC6577492 8182467

[B198] Shadmehr R. Mussa-Ivaldi S. (2012). *Biological Learning and Control: How the Brain Builds Representations, Predicts Events, and Makes Decisions.* Cambridge, MA: The MIT Press.

[B199] Shannon G. F. (1976). A comparison of alternative means of providing sensory feedback on upper limb Prostheses. *Med. Biol. Eng. Comput.* 14 289–294. 10.1007/bf02478123 940388

[B200] Shannon G. F. (1979). A myoelectrically-controlled prosthesis with sensory feedback. *Med. Biol. Eng. Comput.* 17 73–80. 10.1007/bf02440956 312386

[B201] Shehata A. W. Engels L. F. Controzzi M. Cipriani C. Scheme E. J. Sensinger J. W. (2018a). Improving internal model strength and performance of prosthetic hands using augmented feedback. *J. Neuro Eng. Rehabil.* 15 1–12. 10.1186/s12984-018-0417-4 PMC6069837 30064477

[B202] Shehata A. W. Scheme E. J. Sensinger J. W. (2018b). Audible feedback improves internal model strength and performance of myoelectric prosthesis control. *Sci. Rep.* 8 1–10. 10.1038/s41598-018-26810-w 29867147 PMC5986794

[B203] Shehata A. W. Scheme E. J. Sensinger J. W. (2018c). Evaluating internal model strength and performance of myoelectric prosthesis control strategies. *IEEE Trans. Neural Syst. Rehabil. Eng.* 26 1046–1055. 10.1109/TNSRE.2018.2826981 29752240

[B204] Sigrist R. Rauter G. Riener R. Wolf P. (2013). Augmented visual, auditory, haptic, and multimodal feedback in motor learning: a review. *Psychon. Bull. Rev.* 20 21–53. 10.3758/s13423-012-0333-8 23132605

[B205] Simpson D. C. (1972). Externally powered prosthesis for complete arm replacement. *Phys. Med. Biol.* 17 110.

[B206] Simpson D. C. (1974). “The choice of control system for the multimovement prosthesis: extended physiological proprioception,” in *The Control of Upper-Extremity Prostheses and Orthoses*, eds Herberts P. Kadefors R. Magnusson R. Petersen I. (Springfield, IL: Charles Thomas), 146–150.

[B207] Simpson D. C. Smith J. G. (1977). Externally powered controlled complete arm Prosthesis. *J. Med. Eng. Technol.* 1 275–277. 10.3109/03091907709162194 597551

[B208] Soukoreff R. W. MacKenzie I. S. (2004). Towards a standard for pointing device evaluation, perspectives on 27 years of Fitts’ law research in HCI. *Int. J. Hum. -Comput. Stud.* 61 751–789. 10.1016/j.ijhcs.2004.09.001

[B209] Stepp C. E. Matsuoka Y. (2010). “Relative to direct haptic feedback, remote vibrotactile feedback improves but slows object manipulation. 2010,” in *Annual International Conference of the IEEE Engineering in Medicine and Biology*, Piscataway, NJ: IEEE, 2089–2092. 10.1109/IEMBS.2010.5626120 21095683

[B210] Stepp C. E. Matsuoka Y. (2012). Vibrotactile sensory substitution for object manipulation: amplitude versus pulse train frequency modulation. *IEEE Trans. Neural Syst. Rehabil. Eng.* 20 31–37. 10.1109/TNSRE.2011.2170856 21997322 PMC3395369

[B211] Štrbac M. Belić M. Isaković M. Kojić V. Bijelić G. Popović I. (2016). Integrated and flexible multichannel interface for electrotactile stimulation. *J. Neural Eng.* 13:046014 10.1088/1741-2560/13/4/046014 27296902

[B212] Strbac M. Isakovic M. Belic M. Popovic I. Simanic I. Farina D. (2017). Short- and long-term learning of feedforward control of a myoelectric prosthesis with sensory feedback by amputees. *IEEE Trans. Neural. Syst. Rehabil. Eng.* 25 2133–2145. 10.1109/TNSRE.2017.2712287 28600254

[B213] Svensson P. Wijk U. Björkman A. Antfolk C. (2017). A review of invasive and non-invasive sensory feedback in upper limb Prostheses. *Expert Rev Med. Devices* 14 439–447. 10.1080/17434440.2017.1332989 28532184

[B214] Synofzik M. Vosgerau G. Newen A. (2008). Beyond the comparator model: a multifactorial two-step account of agency. *Conscious Cogn.* 17 219–239. 10.1016/j.concog.2007.03.010 17482480

[B215] Szeto A. Y. Saunders F. A. (1982). Electrocutaneous stimulation for sensory communication in rehabilitation engineering. *IEEE Trans. Biomed. Eng.* 29 300–308. 10.1109/tbme.1982.324948 7068167

[B216] Szeto A. Y. J. Lyman J. (1977). Comparison of codes for sensory feedback using electrocutaneous tracking. *Ann. Biomed. Eng.* 5 367–383. 10.1007/BF02367316 607824

[B217] Tan D. W. Schiefer M. A. Keith M. W. Anderson J. R. Tyler J. Tyler D. J. (2014). A neural interface provides long-term stable natural touch perception. *Sci. Transl. Med.* 6:257ra138 10.1126/scitranslmed.3008669 PMC5517305 25298320

[B218] Tan D. W. Schiefer M. A. Keith M. W. Anderson J. R. Tyler J. Tyler D. J. (2014). A neural interface provides long-term stable natural touch perception. *Sci. Trans. Med.* 6:257ra138 10.1126/scitranslmed.3008669 PMC5517305 25298320

[B219] Tejeiro C. Stepp C. E. Ieee M. Malhotra M. Rombokas E. Matsuoka Y. (2012). “Comparison of Remote Pressure and Vibrotactile Feedback for Prosthetic Hand Control,” in *2012 4th IEEE RAS & EMBS International Conference on Biomedical Robotics and Biomechatronics (BioRob)*, Piscataway, NJ: IEEE, 521–525.

[B220] Thumser Z. C. Slifkin A. B. Beckler D. T. Marasco P. D. (2018). Fitts’ law in the control of isometric grip force with naturalistic targets. *Front. Psychol.* 9:560 10.3389/fpsyg.2018.00560 PMC5944157 29773999

[B221] Todorov E. (2004). Optimality principles in sensorimotor control. *Nat. Neurosci.* 7 907–915. 10.1038/nn1309 15332089 PMC1488877

[B222] Todorov E. (2005). Stochastic optimal control and estimation methods adapted to the noise characteristics of the sensorimotor system. *Neural Comput.* 17 1084–1108. 10.1162/0899766053491887 15829101 PMC1550971

[B223] Todorov E. (2006). Optimal control theory. *Environ. Plan. Gov. Policy* 4 1–28. 10.1068/c040121

[B224] Todorov E. (2009). Efficient computation of optimal actions. *Proc. Natl. Acad. Sci. U.S.A.* 106 11478–11483. 10.1073/pnas.0710743106 19574462 PMC2705278

[B225] Todorov E. Jordan M. I. (2002). Optimal feedback control as a theory of motor coordination. *Nat. Neurosci.* 5 1226–1235. 10.1038/nn963 12404008

[B226] Tupper C. N. (1989). Improved prosthesis control. 39–40.

[B227] Tyler D. J. (2016). Restoring the human touch: prosthetics imbued with haptics give their wearers fine motor control and a sense of connection. *IEEE Spectrum* 53 28–33. 10.1109/MSPEC.2016.7459116

[B228] Uno Y. Kawato M. Suzuki R. (1989). Formation and control of optimal trajectory in human multijoint arm movement: minimum torque-change model. *Biol. Cybern.* 61 89–101. 2742921 10.1007/BF00204593

[B229] Valle G. Petrini F. M. Strauss I. Iberite F. D’Anna E. Granata G. (2018b). Comparison of linear frequency and amplitude modulation for intraneural sensory feedback in bidirectional hand Prostheses. *Sci. Rep.* 8:16666 10.1038/s41598-018-34910-w PMC6232130 30420739

[B230] Valle G. Mazzoni A. Iberite F. D’Anna E. Strauss I. Granata G. (2018a). Biomimetic intraneural sensory feedback enhances sensation naturalness, tactile sensitivity, and manual dexterity in a bidirectional prosthesis. *Neuron* 100 37.e7–45.e7. 10.1016/j.neuron.2018.08.033 30244887

[B231] Wan E. A. Van Der Merwe R. (2001). *The Unscented Kalman Filter. Kalman Filtering and Neural Networks.* Hoboken, NJ: John Wiley & Sons, 62.

[B232] Wang G. Zhang X. Zhang J. Gruver W. A. (1995). “Gripping force sensory feedback for a myoelectrically controlled forearm prosthesis,” in *IEEE International Conference on Intelligent Systems for the 21st Century*, Vol. 1 (Vancouver, BC), 501–504.

[B233] Weeks D. L. Wallace S. A. Noteboom J. T. (2000). Precision-grip force changes in the anatomical and prosthetic limb during predictable load increases. *Exp. Brain Res.* 132 404–410. 10.1007/s002210000337 10883390

[B234] Wegner D. (2002). *The Illusion of Conscious Will.* Cambridge, MA: MIT Press.

[B235] Wegner D. (2003). The mind’s best trick: how we experience conscious will. *Trends Cogn. Sci.* 7 65–69. 10.1016/s1364-6613(03)00002-0 12584024

[B236] Wegner D. Sparrow B. (2004). *Authorship Processing. In The new Cognitive Neurosciences.* Cambridge, MA: MIT Press.

[B237] Wegner D. Sparrow B. Winerman L. (2004). Vicarious agency: experiencing control over the movements of others. *J. Pers. Soc. Psychol.* 86 838–848. 10.1037/0022-3514.86.6.838 15149258

[B238] Weir R. F. (1995). *Direct Muscle Attachment as a Control Input for a Position Servo Prosthesis Controller.* Evanston: Northwestern University.

[B239] Weir R. F. Heckathorne C. W. Childress D. S. (2001). Cineplasty as a control input for externally powered prosthetic components. *J. Rehabil. Res. Dev.* 38 357–463. 11563487

[B240] Wettels N. Parnandi A. R. Loeb G. E. Sukhatme G. S. (2009). Grip control using biomimetic tactile sensing systems. *IEEE/ASME Trans. Mech.* 14 718–723. 10.1109/TMECH.2009.2032686

[B241] Wheeler J. Bark K. Savall J. Cutkosky M. (2010). Investigation of rotational skin stretch for proprioceptive feedback with application to myoelectric systems. *IEEE Trans. Neural. Syst. Rehabil. Eng.* 18 58–66. 10.1109/Tnsre.2009.2039602 20071271

[B242] Whitney D. E. (1977). Force feedback control of manipulator fine motions. *J. Dyn. Syst. Measu. Control* 99 91–97. 10.1115/1.3427095

[B243] Witteveen H. J. Rietman H. S. Veltink P. H. (2015). Vibrotactile grasping force and hand aperture feedback for myoelectric forearm prosthesis users. *Prosthetics Orthotics Int.* 39 204–212. 10.1177/0309364614522260 24567348

[B244] Witteveen H. J. B. Droog E. A. Rietman J. S. Veltink P. H. (2012). Vibro- and electrotactile user feedback on hand opening for myoelectric forearm Prostheses. *IEEE Trans. Biomedi. Eng.* 59 2219–2226. 10.1109/TBME.2012.2200678 22645262

[B245] Zafar M. Van Doren C. L. (2000). Effectiveness of supplemental grasp-force feedback in the presence of vision. *Med. Biol. Eng. Comput.* 38 267–274. 10.1007/BF02347046 10912342

[B246] Zollo L. Pino G. Di, Ciancio A. L. Ranieri F. Cordella F. (2019). Restoring tactile sensations via neural interfaces for real-time force-and-slippage closed-loop control of bionic hands. *Sci. Rob.* 4 1–12. 10.1126/scirobotics.aau9924 PMC6795534 31620665

